# Electron Delocalization Realizes Speedy Fenton‐Like Catalysis over a High‐Loading and Low‐Valence Zinc Single‐Atom Catalyst

**DOI:** 10.1002/advs.202304088

**Published:** 2023-10-15

**Authors:** Shaosong Xin, Luning Ni, Peng Zhang, Haobin Tan, Mingyang Song, Tong Li, Yaowen Gao, Chun Hu

**Affiliations:** ^1^ Institute of Environmental Research at Greater Bay Key Laboratory for Water Quality and Conservation of the Pearl River Delta Ministry of Education Guangzhou University Guangzhou 510006 China

**Keywords:** electron delocalization, electron transfer, Fenton‐like catalysis, low valence, zinc single‐atom catalyst

## Abstract

A zinc (Zn)‐based single‐atom catalyst (SAC) is recently reported as an active Fenton‐like catalyst; however, the low Zn loading greatly restricts its catalytic activity. Herein, a molecule‐confined pyrolysis method is demonstrated to evidently increase the Zn loading to 11.54 wt.% for a Zn SAC (Zn_SA_‐N‐C) containing a mixture of Zn−N_4_ and Zn−N_3_ coordination structures. The latter unsaturated Zn−N_3_ sites promote electron delocalization to lower the average valence state of Zn in the mix‐coordinated Zn−N_x_ moiety conducive to interaction of Zn_SA_‐N‐C with peroxydisulfate (PDS). A speedy Fenton‐like catalysis is thus realized by the high‐loading and low‐valence Zn_SA_‐N‐C for PDS activation with a specific activity up to 0.11 min L^−1^ m^−2^, outstripping most Fenton‐like SACs. Experimental results reveal that the formation of Zn_SA_‐N‐C−PDS* complex owing to the strong affinity of Zn_SA_‐N‐C to PDS empowers intense direct electron transfer from the electron‐rich pollutant toward this complex, dominating the rapid bisphenol A (BPA) elimination. The electron transfer pathway benefits the desirable environmental robustness of the Zn_SA_‐N‐C/PDS system for actual water decontamination. This work represents a new class of efficient and durable Fenton‐like SACs for potential practical environmental applications.

## Graphical abstract

Atomically dispersed mix‐coordinated Zn−N_4_ and Zn−N_3_ sites facilitate the formation of Zn_SA_‐N‐C−PDS* complex. Besides a minor superoxide radical (O_2_^●‒^) oxidation route, the electron transfer from the electron‐rich pollutant toward Zn_SA_‐N‐C−PDS^*^ dominates speedy bisphenol A (BPA) remediation.

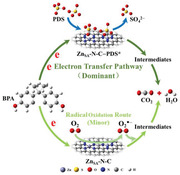

## 1 Introduction

The worldwide water scarcity and increasing need for clean water has provoked the mushroom development of water decontamination technologies over the past decades.^[^ ^1^ ^]^ The Fenton‐like reaction using persulfate, including peroxymonosulfate (PMS) and peroxydisulfate (PDS), as a main oxidant represents a promising strategy for the elimination of refractory organic contaminants, generally thanks to the yield of highly reactive hydroxyl (^●^OH) and/or sulfate (SO_4_^●‒^) radicals upon the dissociation of the O‒O bond in the persulfate molecule.^[^ ^2^, ^3^, ^4^, ^5^ ^]^ Amongst multifarious heterogeneous persulfate activators, carbon‐based single‐atom catalysts (SACs) with utmost atom efficiency, tunable metal coordination, and the confinement effect of electrons have attracted tremendous interest in the Fenton‐like catalysis, particularly in the activation of the asymmetric PMS (H−O−O−SO_3_^−^),^[^ ^6^, ^7^ ^]^ which is considered to be activated more easily compared to PDS with a symmetric structure (SO_3_−O−O−SO_3_^2−^).^[^ ^8^ ^]^

With reference to the reported Fenton‐like SACs, the central metal sources are dominantly concentrated on partially occupied 3d transition‐metals, including cobalt (Co),^[^ ^9^, ^10^, ^11^, ^12^, ^13^, ^14^ ^]^ iron (Fe),^[^ ^15^, ^16^, ^17^, ^18^, ^19^, ^20^ ^]^ manganese (Mn),^[^ ^21^ ^]^ and copper (Cu),^[^ ^22^, ^23^ ^]^ which are intrinsically active for the Fenton‐like chemistry. Aside from those unfilled 3d transition metal atoms, our recent work demonstrated that construction of atomically dispersed zinc‐nitrogen (Zn−N_4_) sites could tune the electron density of the Zn atom and hence convert the inert element Zn with a full‐filled 3d orbital into an active Fenton‐like single‐atom catalyst (SA‐Zn‐NC) for PMS activation, which expanded the variety of Fenton‐like SACs.^[^ ^24^ ^]^ In spite of this, the SA‐Zn‐NC catalyst suffered from a relatively poor Fenton‐like activity due to its low Zn loading (0.82 wt.%). This leaves plenty of room for increase of active site density to enhance the Fenton‐like performance of the Zn‐based SACs. On the other hand, compared to PMS, PDS possesses a higher stability and solubility at room temperature, and meanwhile, causes a lower solution pH decrease at a same concentration.^[^ ^4^, ^25^, ^26^ ^]^ In this context, the SACs‐related PDS activation processes should deserve more attention and investigation. Despite the achievement of a certain progress of PDS activation by SACs, to the best of our knowledge, the activation of PDS over Zn single‐atom catalysts has been rarely reported so far. Moreover, the relevant PDS‐mediated Fenton‐like catalytic mechanism includes radical and/or non‐radical (high‐valent metal‐oxo species, singlet oxygenation, electron transfer) pathways. In particular, the electron transfer route involves the intense interaction of persulfate with catalysts and the subsequent formation of surface‐activated complexes, which triggers the direct electron transport from organic pollutants toward such complexes and thus results in the oxidation of contaminants. This process endows the catalytic systems with high selectivity to electron‐rich organics and remarkable anti‐interference capability to background substances in the complicated water matrix.^[^ ^27^ ^]^ It therefore necessitates an exploration of the electron transfer pathway in the course of PDS activation over Zn SACs.

Herein, we have observably increased the loading of Zn single atom to 11.54 wt.% for a low‐valence Zn‐based SAC (marked as Zn_SA_‐N‐C) via a surface molecule‐confined pyrolysis approach. Different from the common SACs with a single and well‐defined M−N_x_ coordination configuration, the resultant Zn_SA_‐N‐C contains a mixture of atomic Zn−N_4_ and Zn−N_3_ structures. The formation of low‐coordinated Zn−N_3_ sites facilitates electron delocalization and thus affords enough delocalized electrons to lower the oxidation state of the Zn atom conducive to electron transport between the mix‐coordinated Zn−N_x_ moiety and PDS. As a result, the high‐loading and low‐valence Zn_SA_‐N‐C catalyst exhibits a remarkable Fenton‐like performance for the activation of PDS toward bisphenol A (BPA) degradation, with a specific activity up to 0.11 L min^−1^ m^−2^, surpassing most Fenton‐like SACs for PDS and even PMS activation. Detailed experimental results revealed that the strong interaction of Zn_SA_‐N‐C with PDS favors the formation of Zn_SA_‐N‐C−PDS^*^ complex, which triggers direct electron transfer from the electron‐rich organic pollutant toward this adduct, dominating the speedy contaminant oxidation in the course of Fenton‐like catalysis. The electron transfer pathway endows the Zn_SA_‐N‐C/PDS system with high anti‐interference property for the complicated water matrix. Notably, Zn_SA_‐N‐C achieves an efficient pollutant remediation over 216 h when used in a continuous‐flow column reactor, presenting a broad prospect for sustainable environmental applications.

## 2 Results and Discussion

### 2.1 Characterizations of Samples

**Figure 1:**
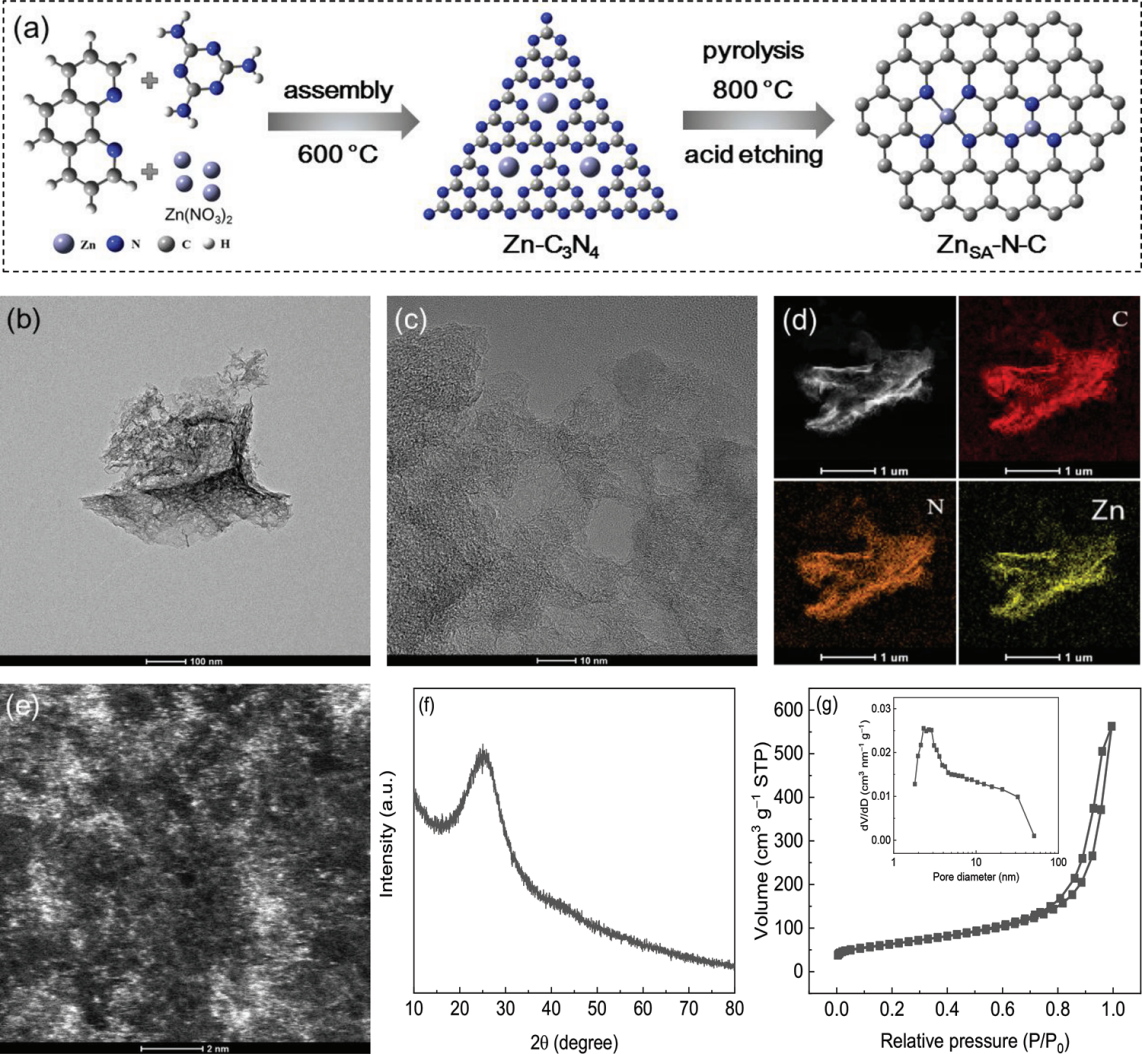
a) Illustration for the synthesis of Zn_SA_‐N‐C. b) TEM, c) HRTEM, and d) elemental mapping images of Zn_SA_‐N‐C. e) HAADF‐STEM image of Zn_SA_‐N‐C. f) XRD pattern and g) N_2_ adsorption/desorption isotherm of Zn_SA_‐N‐C.

The Zn single‐atom catalyst (Zn_SA_‐N‐C) was prepared through a surface molecule‐confined calcination method. As shown in **Figure** **1**a, certain amounts of melamine, phenanthroline and Zn(NO_3_)_2_·6H_2_O are ground uniformly in an agate mortar, where the melamine was utilized as both carbon and nitrogen precursors, and phenanthroline was used as a chelating agent to anchor the Zn species so as to promote the space isolation of Zn ions. The as‐prepared sample was then calcined at 600 °C for 2 h under nitrogen gas atmosphere to yield the Zn‐decorated carbon nitride (Zn‐C_3_N_4_). Upon further pyrolysis at a higher temperature (800 °C), the Zn‐C_3_N_4_ is converted to Zn_SA_‐N‐C after acid etching. The importance of phenanthroline in the synthesis of Zn_SA_‐N‐C can be detailed after Figures S1 and S2 (Supporting Information). The scanning electron microscope (SEM) image (Figure S3, Supporting Information) shows the loose layered and porous structure of Zn_SA_‐N‐C, which is also visualized by the transmission electron microscopy (TEM) analysis (Figure 1b), without any identifiable metallic particles on the surface. The high‐resolution TEM (HRTEM) image (Figure 1c) with the randomly selected region illustrates the typical nature of carbon‐based material for Zn_SA_‐N‐C, and no crystalline Zn species is observed. The Raman spectrum (Figure S4, Supporting Information) further validates the carbonaceous character of Zn_SA_‐N‐C. Elemental mapping by energy‐dispersive X‐ray (EDX) spectroscopy (Figure 1d) manifests the uniform distribution of Zn over the entire N‐doped carbon matrix. The high‐angle annular dark‐field scanning TEM (HAADF‐STEM) image (Figure 1e) clearly reveals the atomic dispersion of Zn atoms in Zn_SA_‐N‐C at a high density, as visualized by numerous bright spots on the surface.^[^ ^28^ ^]^ The Zn content was measured to be 3.04 at% and 11.54 wt.% by XPS and inductively coupled plasma‐optical emission spectroscopy (ICP‐OES) (Table S1, Supporting Information), respectively, suggesting a high loading of Zn for Zn_SA_‐N‐C. The atomically dispersed Zn species can be also evidenced by the X‐ray diffraction (XRD) pattern of Zn_SA_‐N‐C (Figure 1f) which shows only diffraction from graphitic carbon but no Zn‐related crystalline phase. The N_2_ adsorption/desorption isotherm (Figure 1g) exhibits a representative type IV curve for Zn_SA_‐N‐C, with its specific surface area of 222.3 m^2^ g^−1^. The relevant pore size distribution curve (inset of Figure 1g) indicates the mesoporous structure of Zn_SA_‐N‐C.

**Figure 2:**
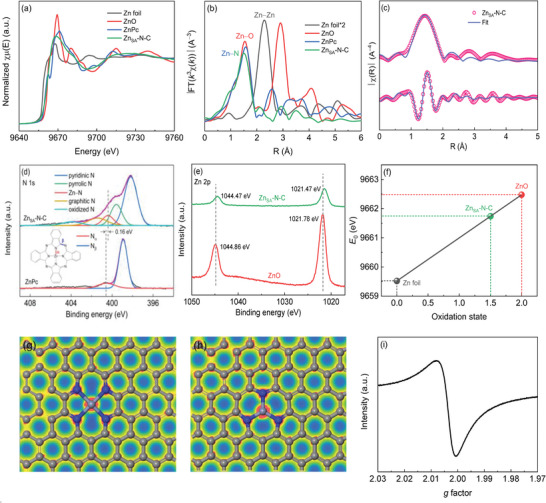
a) Zn K‐edge XANES and b) Fourier transformed EXAFS spectra of Zn_SA_‐N‐C and references. c) EXAFS fitting curves of Zn_SA_‐N‐C using ZnPc as a well‐defined Zn−N_4_ core structure in R space. XPS d) N 1s and e) Zn 2p spectra of Zn_SA_‐N‐C and reference samples. f) Average oxidation state of Zn in Zn_SA_‐N‐C and references based on the absorption threshold energy (*E*_0_) obtained from the first differentiation. Electron density distribution of g) Zn‒N_4_ and h) Zn‒N_3_ structures (cyan, blue, and gray spheres represent Zn, N, and C atoms, respectively). i) Solid EPR spectra of Zn_SA_‐N‐C.

The X‐ray absorption spectroscopy (XAS) analysis was performed to probe the coordination structure and valence state of the Zn atom in Zn_SA_‐N‐C. The Zn K‐edge X‐ray absorption near‐edge structure (XANES) spectra (**Figure** **2**a) demonstrate that the absorption edge position of Zn_SA_‐N‐C locates away from that of Zn foil but approaches that of zinc phthalocyanine (ZnPc), and meanwhile, is lower than that of ZnO. This implies that the Zn atom is in an oxidation state between Zn^0^ and Zn^2+^. Similarly, the white line peak intensity of Zn_SA_‐N‐C is higher than that of Zn foil, while is inferior to that of ZnPc and ZnO. The ZnPc molecule possesses a typical Zn−N_4_ configuration, more specially, the Zn−pyrrolic N_4_ structure (Figure S5, Supporting Information). In the XANES spectrum of ZnPc, the *D*_4h_ symmetry fingerprint peak of Zn−pyrrolic N_4_ appears at ≈9662.4 eV. However, this symmetry peak disappears in the spectrum of Zn_SA_‐N‐C, which suggests that Zn_SA_‐N‐C contains no Zn−pyrrolic N_4_ moiety, and that a non‐centrosymmetric Zn−N structure may form in Zn_SA_‐N‐C.^[^ ^29^, ^30^ ^]^ From the Fourier transformed extended X‐ray absorption fine structure (EXAFS) spectra (Figure 2b), Zn_SA_‐N‐C shows only one primary peak at 1.50 Å assignable to the Zn−N scatting path, without the detection of Zn−Zn (at 2.28 Å) and second‐shell Zn−O−Zn (at 2.91 Å) bonds.^[^ ^24^, ^31^ ^]^ Besides, the wavelet transform (WT) analysis of Zn_SA_‐N‐C and reference samples was further conducted. As shown in Figure S6 (Supporting Information), the WT contour plots of Zn_SA_‐N‐C exhibits one intensity maximum at ≈5.65 Å^−1^, which is close to that in ZnPc (6.15 Å^−1^) attributable to the Zn−N coordination. Moreover, the signals derived from the Zn−Zn contribution in the Zn foil (7.35 Å^−1^) and ZnO (7.25 Å^−1^) are unobservable in Zn_SA_‐N‐C. These results reveal that the Zn element exists in the nitrogen‐coordinated atomically dispersed form in Zn_SA_‐N‐C. In the spectrum of ZnPc containing a well‐defined Zn−N_4_ configuration, the Zn−N bond length is 1.57 Å. By contrast, Zn_SA_‐N‐C delivers a slightly shorter Zn−N interatomic distance of 1.50 Å, implying a contractive Zn−N local coordination center in Zn_SA_‐N‐C.^[^ ^30^ ^]^ In the meantime, the Zn−N peak intensity of Zn_SA_‐N‐C is also weaker relative to that of ZnPc, which indicates the coordination number of Zn around N in Zn_SA_‐N‐C is <4.^[^ ^12^, ^32^ ^]^ Considering the ZnPc molecule possesses a representative Zn−N_4_ configuration, ZnPc was used as a Zn−N_4_ core structure for the EXAFS fitting to ascertain the local coordination of Zn_SA_‐N‐C. According to the first‐shell EXAFS fitting results (Figure 2c; Figure S7 and Table S2, Supporting Information), the average coordination number of Zn with N in Zn_SA_‐N‐C is found to be 3.6, which is lower than that in ZnPc (3.9). The lower Zn−N coordination number means that, besides the normal Zn−N_4_ moiety, an unsaturated Zn−N_x_ (x < 4) structure may also form in Zn_SA_‐N‐C. In this context, a lower‐coordinated Zn−N_3_ model was constructed via density functional theory (DFT) calculations (Figure S8a, Supporting Information) and utilized as a core structure to conduct the EXAFS fitting for Zn_SA_‐N‐C. However, the relevant fitting results turn out to be less satisfactory due to the larger *R*‐factor (percentage misfit) and the unmatched coordination number (Figure S8b and Table S3, Supporting Information), indicating Zn_SA_‐N‐C is not just composed of the Zn−N_3_ configuration. The above results demonstrate that Zn_SA_‐N‐C consists of a mixture of Zn−N_4_ and Zn−N_3_ coordination structures.^[^ ^30^ ^]^

In addition, the XPS analysis was further performed to investigate the chemical structure of Zn_SA_‐N‐C. As displayed in Figure 2d, the XPS N 1s spectrum of ZnPc can be deconvoluted into two N_α_ and N_β_ sub‐peaks, the former of which refers to the Zn−pyrrolic N bond in the Zn−pyrrolic N_4_ structure.^[^ ^33^ ^]^ The Zn_SA_‐N‐C contains five peaks corresponding to pyridinic N, pyrrolic N, Zn−N, graphitic N, and oxidized N. Notably, the position of Zn−N peak in Zn_SA_‐N‐C is close to that in ZnPc but with a negative shift of 0.16 eV. This result suggests the existence of Zn−N_4_ configuration in Zn_SA_‐N‐C, while the Zn atom may coordinate with other N species rather than pyrrolic N. The absence of Zn−C peak in the XPS C 1s spectrum (Figure S9, Supporting Information) excludes the bonding of Zn and C atoms in Zn_SA_‐N‐C. As the organic precursors would decompose completely at elevated pyrolysis temperature, the calcination of the mixture of melamine and phenanthroline (without the Zn source) with the same method to the preparation of Zn_SA_‐N‐C cannot produce the pure nitrogen‐doped carbon (N‐C) material. To determine which nitrogen species, namely pyridinic N or pyrrolic N, is more prone to be bonded with the Zn atom to form atomic Zn−N_4_ and Zn−N_3_ structures, the formation energies (*E*_f_) of different Zn−N_x_ configurations were calculated by density functional theory (DFT) computations and then compared. As shown in Figure S10 (Supporting Information), the Zn−pyridinic N_4_ configuration delivers a much lower formation energy than the Zn−pyrrolic N_4_ one. Similarly, the formation energy of the Zn−pyridinic N_3_ model is inferior to that of the Zn−pyrrolic N_3_ one. The lower *E*_f_ values of Zn−pyridinic N_x_ structures relative to those of Zn−pyrrolic N_x_ ones suggest that the isolated Zn atoms are more likely to bond with pyridinic N to constitute Zn−pyridinic N_4_ and Zn−pyridinic N_3_ configurations in Zn_SA_‐N‐C. Thus, the atomic structure of Zn_SA_‐N‐C containing a mixture of Zn−pyridinic N_4_ and Zn−pyridinic N_3_ moieties can be visualized in Figure 1a above. In the XPS Zn 2p spectra (Figure 2e), the Zn 2p_3/2_ and Zn 2p_1/2_ peaks in Zn_SA_‐N‐C are situated at lower binding energies compared with those in the ZnO reference, reflecting a low oxidation state (<+2) of Zn for Zn_SA_‐N‐C. To quantify the oxidation state of Zn species, the correlation between Zn valence state and the absorption threshold energy (*E*_0_) obtained from the first differentiation in the main absorption edge region of XANES was explored. As depicted in Figure 2f, the average oxidation state of Zn in Zn_SA_‐N‐C is determined to be ≈+1.50. The decrease of average valence state of Zn can be attributable to the formation of low‐coordinated Zn−N_3_ configuration. The electron density distribution analysis (Figure 2g,h) illuminates electron accumulation around the Zn atom in both Zn−N_4_ and Zn−N_3_ models. Specifically, the Hirshfeld charge of the Zn atom was calculated to be 0.405 and 0.390 e in Zn−N_4_ and Zn−N_3_ models, respectively. A lower Hirshfeld charge signifies an electron‐richer environment of the Zn atom in the unsaturated Zn−N_3_ configuration, suggesting that the formed Zn−N_3_ structure holds abundant delocalized electrons to reduce the average valence state of Zn in Zn_SA_‐N‐C. The electron delocalization of the mix‐coordinated Zn−N_x_ (particularly Zn−N_3_) sites can be evidenced by a strong signal with a *g*‐factor of 2.003 referring to free electrons in the solid‐state electron paramagnetic resonance (EPR) spectrum of Zn_SA_‐N‐C (Figure 2i). All the above characterization results manifest that Zn_SA_‐N‐C contains a non‐centrosymmetric mix‐coordination of single‐atomic and high‐density Zn−N_4_ and Zn−N_3_ structures, which results in the decrease of the average valence of the Zn atom. Benefiting from the high Zn loading to provide abundant Zn−N_x_ sites and the electron delocalization of Zn−N_x_ centers to facilitate interfacial electron transfer, the Zn_SA_‐N‐C catalysts is expected to deliver an admirable Fenton‐like activity.

### 2.2 Fenton‐Like Performance of Zn_SA_‐N‐C

**Figure 3:**
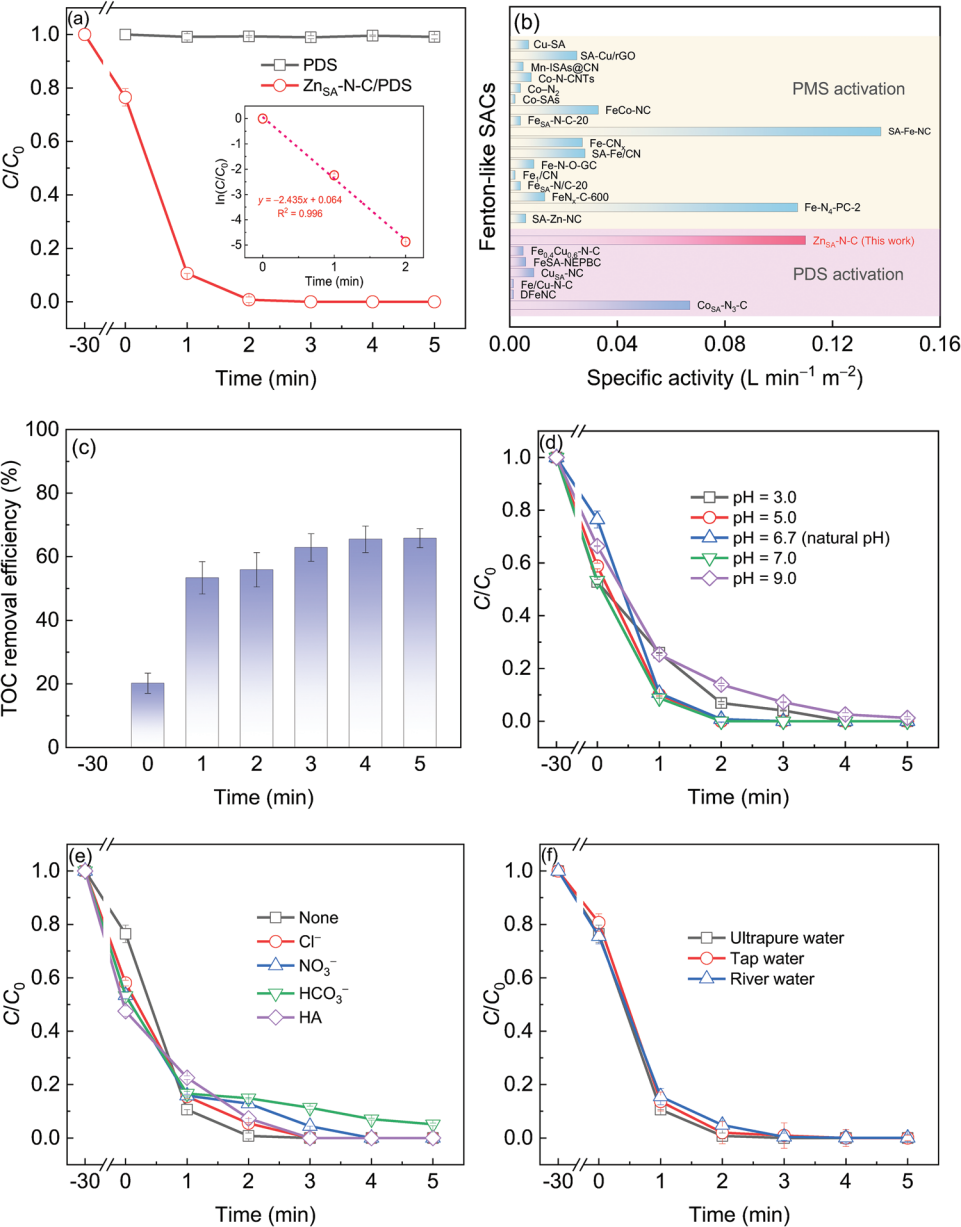
a) Fenton‐like activity of Zn_SA_‐N‐C. b) Specific activity comparison of Zn_SA_‐N‐C with currently reported transition‐mental‐based SACs for PDS and even PMS activation. c) TOC removal as a function of reaction time. Effects of d) initial pH and e) common environmental‐relevant substances on BPA removal by Zn_SA_‐N‐C with PDS. f) BPA removal in different water systems. Reaction conditions: [BPA] = 100 µM; [Zn_SA_‐N‐C] = 0.1 g L^‒1^; [PDS] = 2 mM; [Temp] = 30°C; initial pH = 6.7; [Cl^‒^] = [HCO_3_^‒^] = [NO_3_^‒^] = 1 mM; [HA] = 10 mg L^‒1^. (if any).

The Fenton‐like performance of Zn_SA_‐N‐C was assessed by catalytic oxidation of bisphenol A (BPA, a typical emerging contaminant in water) in the presence of PDS. As displayed in **Figure** **3**a, a 30‐min pre‐interaction of Zn_SA_‐N‐C with BPA causes ≈24% of BPA removal. The PDS alone affords an insignificant removal of BPA, indicating the incapability of PDS in oxidizing BPA directly. However, the addition of PDS into the suspension brings about the complete BPA elimination within 3 min, suggesting a superb Fenton‐like reactivity of Zn_SA_‐N‐C. According to pseudo‐first‐order kinetics (ln(*C*/*C*_0_) = −*kt*, inset of Figure 3a), the reaction rate constant of Zn_SA_‐N‐C for PDS activation toward BPA oxidation was found to be 2.44 min^−1^. By normalizing the rate constant to specific surface area and dosage of the catalyst, the specific activity of Zn_SA_‐N‐C was calculated to be 0.11 L min^−1^ m^−2^ (Table S4, Supporting Information), which is much greater than that of most Fenton‐like SACs for PDS and even PMS activation (Figure 3b), reflecting that Zn_SA_‐N‐C is among the rank of excellent Fenton‐like SACs. The increase of total organic carbon (TOC) removal efficiency as a function of reaction time (Figure 3c) attests to the degradation of BPA by Zn_SA_‐N‐C via PDS activation. It is worth noting that the pre‐interaction between Zn_SA_‐N‐C and BPA for 30 min produces ≈20% of TOC removal, which is slightly lower than the BPA removal efficiency (≈24%), suggesting that besides BPA adsorption, the oxidation of BPA occurs over Zn_SA_‐N‐C simultaneously (see more discussions in the next section). The initial pH exerts an insignificant impact on the abatement of BPA (Figure 3d), illustrating the effectiveness of Zn_SA_‐N‐C/PDS over a broad pH range. The inappreciable influence of common environmental‐relevant substances including inorganic anions and natural organic matter such as humic acid (HA) on BPA decay (Figure 3e) reveals the sound tolerance of the Zn_SA_‐N‐C/PDS system to the complicated water matrix. Moreover, the efficient elimination of BPA can be fulfilled in tap and river waters (Figure 3f) (their water quality parameters could be available from our previous work),^[^ ^24^ ^]^ which manifests the potential practicability of Zn_SA_‐N‐C for actual environmental remediation with PDS.

### 2.3 Mechanistic Insights into Fenton‐Like Catalysis

**Figure 4:**
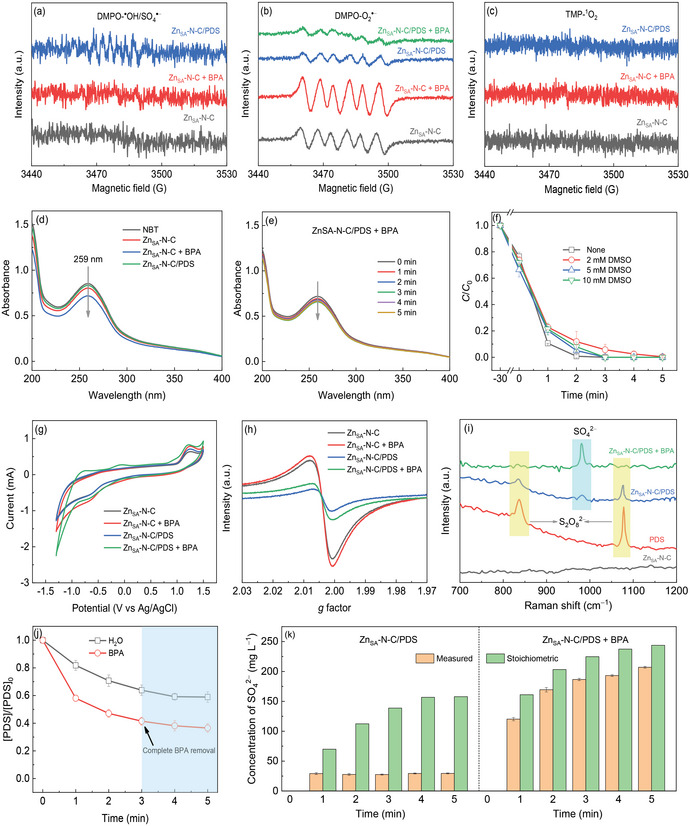
EPR spectra for a) ^●^OH/SO_4_^●‒^, b) O_2_^●‒^ and c) ^1^O_2_ of different systems. d) Absorption spectra of NBT catalyzed by Zn_SA_‐N‐C with and without BPA or PDS for 30 min. e) Time‐resolved absorption spectra of NBT catalyzed by Zn_SA_‐N‐C with PDS in the presence of BPA. f) Effect of DMSO on BPA removal by Zn_SA_‐N‐C/PDS. g) CV curves, and h) solid EPR and i) in situ Raman spectra of Zn_SA_‐N‐C in different conditions. j) Decay of PDS over Zn_SA_‐N‐C with and without BPA. k) Residual SO_4_^2‒^ concentration in different systems with reaction time.

The PDS‐mediated Fenton‐like catalysis generally involves the formation of reactive oxygen species (ROS). In the course of PDS activation by Zn_SA_‐N‐C, the yield of ROS was detected via the EPR‐ measurements. As shown in **Figure** **4**a, neither ^●^OH nor SO_4_^●‒^ signals are visible for Zn_SA_‐N‐C irrespective of the addition of BPA or PDS by using 5,5‐dimethyl‐1‐pyrroline N‐oxide (DMPO) as a spin‐trapping reagent, excluding the generation of ^●^OH and SO_4_^●‒^ during the interaction between Zn_SA_‐N‐C and BPA or PDS. In contrast, the characteristic signal of superoxide radical (O_2_^●‒^) can be clearly noted for the Zn_SA_‐N‐C alone using DMPO as a trapping agent (Figure 4b). After the addition of benzoquinone (BQ, a typical scavenger for O_2_^●‒^), the O_2_^●‒^ peak intensity experiences a dramatic decrease for bare Zn_SA_‐N‐C (Figure S11, Supporting Information). Upon N_2_ aeration, the O_2_^●‒^ signal vanishes completely for the individual Zn_SA_‐N‐C. These phenomena illustrate the capability of Zn_SA_‐N‐C in dissolved oxygen (DO) activation for O_2_^●‒^ production. Intriguingly, the intensity of O_2_^●‒^ signal increases after the introduction of BPA into the Zn_SA_‐N‐C suspension (Figure 4b), which implies that the presence of BPA boosts DO reduction over Zn_SA_‐N‐C to produce more amount of O_2_^●‒^. Nonetheless, the replacement of BPA with PDS decreases the O_2_^●‒^ peak intensity, indicating that PDS weakens the reduction of DO by Zn_SA_‐N‐C. Meanwhile, the infusion of BPA into the Zn_SA_‐N‐C/PDS system further attenuates the intensity of O_2_^●‒^ peak, which can be ascribed to the BPA oxidation by a small amount of O_2_^●‒^. Similarly with ^●^OH and SO_4_^●‒^, the non‐radical species, namely singlet oxygen (^1^O_2_), is undetectable for Zn_SA_‐N‐C in the absence and presence of BPA or PDS, as evidenced by the inexistence of any ^1^O_2_ signal in different suspensions with 2,2,6,6‐tetramethyl‐4‐piperidinol (TMP) (Figure 4c). The quenching tests (Figure S12a, Supporting Information) suggest that the injection of isopropanol (IPA, a scavenger for ^●^OH) or ethanol (a quencher for SO_4_^●‒^) exerts an insignificant suppression on BPA oxidation over Zn_SA_‐N‐C with PDS, while the presence of BQ shows a slight inhibition on BPA degradation in Zn_SA_‐N‐C/PDS. As for ^1^O_2_, the addition of TMP (a typical ^1^O_2_ quencher) with different concentrations hardly alters the degradation of BPA (Figure S12b, Supporting Information). Nonetheless, when furfuryl alcohol (FFA) was used as another ^1^O_2_ quenching agent, the BPA abatement is slightly inhibited at a FFA concentration of 5 mm (Figure S12c, Supporting Information), which is contradictory to above EPR and quenching test results in terms of no ^1^O_2_ formation. Guan et al.^[^ ^34^ ^]^ reported the analogous inhibitory effect of FFA on organic pollutant degradation during PDS activation by carbon nanotube (CNT). They demonstrated that the high‐concentration FFA could competitively consume the reactive nonradical PDS−CNT species leading to the inhibition of bromophenol oxidation. Moreover, Ren et al.^[^ ^35^ ^]^ also observed that FFA profoundly inhibited the oxidation of phenolic compounds via suppressing the electron transfer between the radical‐free CNT−PDS^*^ complex and phenolic pollutants, without the production of ^1^O_2_. According to these literatures, the FFA concentration was increased to probe its impact on BPA degradation by the present Zn_SA_‐N‐C/PDS system. As expected, FFA at a higher concentration of 10 mm evidently restrains the degradation of BPA (Figure S12c, Supporting Information). This result rules out the formation of ^1^O_2_ during PDS activation by Zn_SA_‐N‐C, and furthermore, implies the existence of electron transfer pathway behind the Fenton‐like oxidation of BPA by Zn_SA_‐N‐C.

Without the involvement of ^1^O_2_, the substitution of deuteroxide (D_2_O) for H_2_O exhibits an insignificant influence on the overall BPA oxidation in Zn_SA_‐N‐C/PDS (Figure S13, Supporting Information). However, the BPA oxidation performance in D_2_O is slightly slower compared to that in H_2_O within the reaction time from 1 to 3 min. This phenomenon is similar with a recent work reporting that the replacement of H_2_O with D_2_O decreased PMS decomposition and hence retarded PMS activation, giving rise to the D_2_O exchange‐induced attenuation of 4‐chlorophenol (4‐CP) degradation in the annealed diamonds (AND)/PMS system.^[^ ^36^ ^]^ In view of the similarity of PMS and PDS, it can be assumed that the substitution of H_2_O with D_2_O could also weaken the decomposition of PDS by Zn_SA_‐N‐C. Following this literature, the PDS decay over Zn_SA_‐N‐C in D_2_O was explored. As depicted in Figure S14a (Supporting Information), the decay of PDS is indeed slower in D_2_O as compared with that in H_2_O. Considering the existence of BPA can boost PDS decomposition by Zn_SA_‐N‐C apparently (see Figure 4j below), the consumption of PDS in D_2_O with the presence of BPA was also investigated. From Figure S14b (Supporting Information), the PDS consumption in D_2_O is also inferior to that in H_2_O. Therefore, the lower BPA degradation in D_2_O than that in H_2_O can be attributable to the retardation of PDS activation by D_2_O. The above EPR measurement, quenching test, and solvent exchange experiment results illuminate no generation of ^●^OH/SO_4_^●‒^ and ^1^O_2_, but the production of a handful of O_2_^●‒^ during PDS activation over Zn_SA_‐N‐C.

To validate the yield of O_2_^●‒^ over Zn_SA_‐N‐C, the nitroblue tetrazolium (NBT) method was adopted since O_2_^●‒^ can interact with NBT to weaken the maximum absorbance of NBT at 259 nm.^[^ ^37^, ^38^ ^]^ As depicted in Figure 4d, the maximum absorption intensity of NBT undergoes a decrease after interacting with Zn_SA_‐N‐C for 30 min, confirming the O_2_^●‒^ generation over Zn_SA_‐N‐C via DO reduction. The attenuation of NBT absorbance continues to proceed upon the addition of BPA into the Zn_SA_‐N‐C suspension, while the infusion of PDS reduces the NBT absorption intensity, which symbolizes the promotion and demotion of DO reduction over Zn_SA_‐N‐C by BPA and PDS, respectively. The further introduction of PDS into the suspension of Zn_SA_‐N‐C and BPA decreases the NBT maximum absorbance slightly (Figure 4e), which suggests the limited production of O_2_^●‒^ in the system of Zn_SA_‐N‐C/PDS + BPA, consistent with the relevant EPR analysis in Figure 4b above. Considering the capacity of Zn_SA_‐N‐C in molecular oxygen activation for O_2_^●‒^ formation, the BPA removal by the single Zn_SA_‐N‐C was investigated. As displayed in Figure S15a (Supporting Information), the augment of the catalyst dosage from 0.05 to 0.2 g L^−1^ produces a finite enhancement for BPA elimination by the Zn_SA_‐N‐C alone after a 30‐min reaction, implying that the O_2_^●‒^ generated from DO reduction over Zn_SA_‐N‐C seems to be insufficient to oxidize BPA efficiently. In this regard, the desirable BPA degradation necessitates the introduction of PDS. As anticipated, the addition of PDS apparently accelerates the oxidation of BPA, and the greater dosage of Zn_SA_‐N‐C affords the higher BPA degradation kinetics (Figure S15a, Supporting Information) and the larger reaction rate constant (Figure S15b, Supporting Information). Nevertheless, these reinforcements become less significant by increasing the catalyst dose from 0.1 to 0.2 g L^−1^. Unlike the apparent rate constant, the specific activity first increases from the catalyst loading of 0.05 to 0.1 g L^−1^, but decreases with the further augment to 0.2 g L^−1^ (Figure S15c, Supporting Information). This phenomenon may be explained by the following reasons. On one hand, the augment of catalyst dosage can assuredly provide more active sites to strengthen the interaction between Zn_SA_‐N‐C and reactants, so as to enhance the apparent activity of Zn_SA_‐N‐C. On the other hand, the higher dose of the catalyst is likely to impede its complete uniform suspension in the reaction solution, which cannot guarantee the full accessibility of active sites per surface area to reactants, giving rise to the decrease of specific activity of Zn_SA_‐N‐C at the greater catalyst loading. In addition, the larger dosage of Zn_SA_‐N‐C causes more serious leaching of Zn ions (Figure S16, Supporting Information), and meanwhile, increases the resource consumption, particularly for the large‐scale application. Taking above aspects together, the optimum catalyst dosage is selected to be 0.1 g L^−1^ to fulfill the upmost specific activity with resource consumption and Zn leaching as low as possible.

Given the only generation of a small quantity of O_2_^●‒^ (without other ROS) during PDS activation by Zn_SA_‐N‐C, the rapid BPA degradation in the Zn_SA_‐N‐C/PDS system may be mainly originated from the non‐radical pathway including high‐valence metal species formation and electron transfer process. The influence of dimethyl sulfoxide (DMSO) on BPA oxidation was explored to probe the formation of high‐valent Zn species. As shown in Figure 4f, the presence of DMSO with different concentrations shows an inappreciable impact on catalytic degradation of BPA, suggesting no formation of high‐valence Zn species in the course of PDS activation by Zn_SA_‐N‐C. In this case, the electron transfer pathway is likely to be responsible for the speedy BPA oxidation during the Fenton‐like catalysis. In cyclic voltammetry (CV) curves (Figure 4g), the Zn_SA_‐N‐C electrode delivers two main peaks at ≈−0.65 and 1.24 V corresponding to the reduction of DO and the oxidation of Zn species, respectively. After adding BPA into the electrolyte, both DO reduction and Zn species oxidation are enhanced for Zn_SA_‐N‐C, suggesting that the electron transfer between Zn_SA_‐N‐C and BPA facilitates DO reduction over Zn_SA_‐N‐C. By contrast, the presence of PDS exhibits an insignificant influence on DO reduction but a slight enhancement for Zn species oxidation compared to the Zn_SA_‐N‐C alone, which implies a mild electron transport from Zn_SA_‐N‐C toward PDS. Upon the co‐existence of PDS and BPA, the redox currents are intensified, signifying that BPA boosts the interfacial electron transfer between Zn_SA_‐N‐C and reactants. From the solid‐state EPR spectra (Figure 4h), the increased signal associated with free electrons for Zn_SA_‐N‐C after reacting with BPA illuminates the electron delivery from BPA to Zn_SA_‐N‐C. On the contrary, such peak intensity decreases after the interaction between Zn_SA_‐N‐C and PDS, which may be attributable to the binding of PDS onto single‐atomic Zn−N_x_ sites, as affirmed by a pronounced inhibition of BPA oxidation upon the addition of phenanthrolin, a strong chelating agent for the metal species,^[^ ^24^ ^]^ into the suspension of Zn_SA_‐N‐C and PDS (Figure S17, Supporting Information). In the meantime, a slight electron transport from Zn_SA_‐N‐C toward PDS also takes place. After further interaction of BPA with Zn_SA_‐N‐C and PDS, the paramagnetic signal in turn increases to some extent, indicating that the organic compound transfers the electron toward the PDS‐attached Zn_SA_‐N‐C.

The strong interaction of Zn_SA_‐N‐C and PDS can be confirmed by the in situ Raman analysis. As shown in Figure 4i, the pure PDS solution shows two primary peaks at ≈836 and 1076 cm^−1^.^[^ ^35^ ^]^ After interacting with Zn_SA_‐N‐C, these two peaks appear in the spectrum of Zn_SA_‐N‐C/PDS, suggesting the strong adsorption of PDS on the surface of the catalyst. Notably, the low‐wave‐number PDS peak downshifts slightly (Figure S18, Supporting Information), which testifies to the formation of a complex between Zn_SA_‐N‐C and PDS (Zn_SA_‐N‐C−PDS^*^).^[^ ^39^ ^]^ The increase of the open‐circuit potential for Zn_SA_‐N‐C after contact with PDS further corroborates that the Zn_SA_‐N‐C−PDS^*^ complex forms during PDS activation by Zn_SA_‐N‐C owing to the higher oxidation potential of Zn_SA_‐N‐C−PDS^*^ as compared to that of Zn_SA_‐N‐C (Figure S19, Supporting Information).^[^ ^39^, ^40^ ^]^ Besides such two peaks, a new weak peak emerges at 982 cm^−1^ in the Raman spectrum of Zn_SA_‐N‐C/PDS, which is accredited to the sulfate anion (SO_4_^2−^).^[^ ^41^, ^42^ ^]^ This means that both a strong PDS adsorption and a slight PDS decomposition occur in the course of PDS activation over Zn_SA_‐N‐C. After the injection of BPA in the Zn_SA_‐N‐C/PDS suspension, the original PDS peaks nearly disappear, whereas the SO_4_^2−^ peak intensity augments evidently, illustrating that the addition of BPA significantly facilitates PDS decomposition to yield SO_4_^2−^ directly. The promotion of BPA for the decomposition of PDS can be ascertained by a remarkably enhanced PDS decay over Zn_SA_‐N‐C in the presence of BPA (Figure 4j). In the meanwhile, the decay of PDS becomes slowly after the complete removal of BPA. As is well‐known, the direct conversion of PDS into SO_4_^2−^ should proceed a 2e^−^ PDS reduction reaction, as expressed by Equation (1).

(1) $$S_{2}O_{8}{{}^{2}}^{-}+2e^{-}\to 2{\mathrm{SO}}_{4}{{}^{2}}^{-}$$

To verify the BPA‐induced two‐electron transfer pathway for PS activation by Zn_SA_‐N‐C, the concentrations of residual SO_4_^2−^ with and without BPA were determined by an ion chromatography (IC). From Figure 4k, in the absence of BPA, the measured SO_4_^2−^ concentrations are far below the stoichiometric amounts calculated from Equation (1) according to the time course decay of PDS on Zn_SA_‐N‐C. This reflects that PDS adsorption onto Zn_SA_‐N‐C mainly accounts for the decline of PDS concentration, though accompanied by a weak PDS decomposition over the catalyst. In the presence of BPA, the measured concentrations of SO_4_^2−^ are close to the stoichiometric ones, suggesting that the electron transfer from BPA toward Zn_SA_‐N‐C−PDS^*^ affords the direct PDS conversion to SO_4_^2−^, along with the speedy oxidation of BPA itself. It should be noted that the actual residual SO_4_^2−^ concentration after the Fenton‐like reaction was measured to be 207 mg L^−1^, which is below the permissible limit (250 mg L^‒1^) in drinking water based on the Chinese National Standard (GB 3838‐2002).

**Figure 5:**
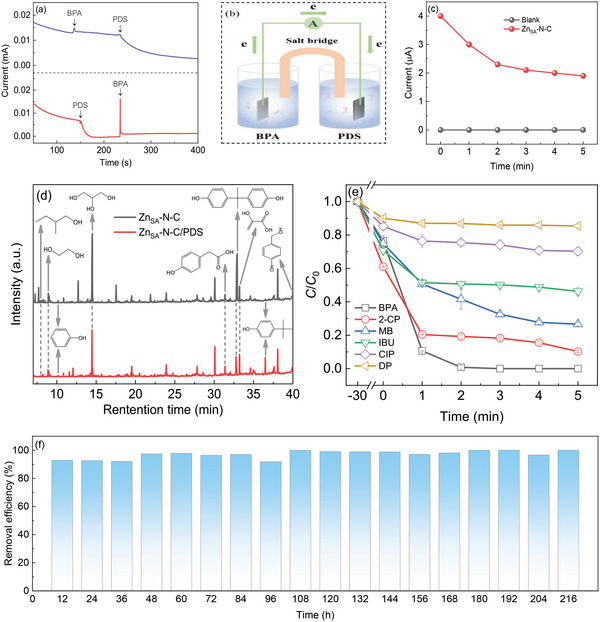
a) Current response of the Zn_SA_‐N‐C electrode after adding BPA and PDS in different sequences. b) The two‐cell device setup of GOS. c) Current generation in GOS coating with Zn_SA_‐N‐C. d) GC‐MS chromatogram of BPA elimination by Zn_SA_‐N‐C after reaction for 30 min (up) and Zn_SA_‐N‐C/PDS after reaction for 1 min (down). e) Removal of various organic pollutants by Zn_SA_‐N‐C with PDS. f) Long‐term BPA elimination by a homemade continuous‐flow column reactor. Reaction conditions: [pollutant] = 100 µM; [Zn_SA_‐N‐C] = 0.1 g L^‒1^; [PDS] = 2 mM; [Temp] = 30°C; initial pH = 6.7 for (d) and (e). [BPA] = 100 µM; [Zn_SA_‐N‐C] = 0.15 g; [PDS] = 2 mM; [flowing rate] = 2 mL min^−1^; [hydraulic retention time, HRT] = 25 min for (f).

The intense electron migration between BPA and Zn_SA_‐N‐C−PDS^*^ was also explored by recording the current response for the Zn_SA_‐N‐C electrode upon the addition of reactants in different sequences. As depicted in **Figure** **5**a, the first injection of BPA results in a slight increase of the current, confirming that BPA conveys the electron to Zn_SA_‐N‐C. The subsequent introduction of PDS in turn reduces the electrode current, which validates the affinity of single‐atomic Zn−N_x_ sites to PDS and the electron delivery from Zn_SA_‐N‐C toward PDS. The analogous trend is noted when PDS was added preferentially. Afterward, the infusion of BPA produces a sharp augment for the current, substantiating the strong electron donation from BPA to the Zn_SA_‐N‐C−PDS* complex, which is conducive to BPA oxidation. In this context, without the interaction between Zn_SA_‐N‐C and BPA in advance, the simultaneous addition of PDS and Zn_SA_‐N‐C into the BPA solution offers a slightly higher BPA oxidation kinetics compared to that with pre‐interaction of Zn_SA_‐N‐C and BPA for 30 min (Figure S20, Supporting Information). A galvanic oxidation system (GOS) with a two‐cell configuration connected by a salt bridge was constructed to further ascertain the direction of electron transfer.^[^ ^43^ ^]^ The device setup is illustrated in Figure 5b, where the two cells contain BPA and PDS solutions, respectively. As displayed in Figure 5c, no device current can be observed on a multimeter with graphite electrodes being immersed into two solutions. In contrast, an obvious current is recorded by substituting the Zn_SA_‐N‐C‐coated graphite electrode for the primitive graphite electrode in the PDS solution. The direction of current substantiates that electrons are transferred from BPA toward PDS through the Zn_SA_‐N‐C catalyst.^[^ ^43^ ^]^ The above results demonstrate that, besides a minor radical (O_2_^●‒^) oxidation route, the electron transfer pathway via the formation of Zn_SA_‐N‐C−PDS^*^ to abstract electrons from BPA is dominantly responsible for the speedy BPA degradation during the Fenton‐like catalysis over Zn_SA_‐N‐C. The detection of several intermediates via gas chromatography‐mass spectrometer (GC‐MS) (Figure 5d; Figure S21, Supporting Information) verifies the occurrence of partial BPA degradation during the interaction between BPA and Zn_SA_‐N‐C. The succedent introduction of PDS leads to an evident decrease of BPA peak intensity, and meanwhile, an obvious peak associated with 4‐tert‐butylphenol (retention time at ≈36.5 min) appears. This signifies that the addition of PDS expedites the oxidation of BPA over Zn_SA_‐N‐C mainly via an electron transfer route. On such basis, the possible BPA degradation pathway is proposed in Figure S22 (Supporting Information). To be specific, the BPA molecule first loses electrons to break up into 4‐tert‐butylphenol and phenol, which are further oxidized and decomposed into other aromatic compounds and small molecular alcohols and acids. The in situ diffuse reflectance infrared Fourier transform spectroscopy (DRIFTS) analysis (Figure S23, Supporting Information) further supports the degradation of BPA into the phenolic compounds and carboxylic acids as well as alcohols during the PDS activation over Zn_SA_‐N‐C (see more details after Figure S23, Supporting Information). However, the detailed decomposition of intermediate phenolic compounds to alcohols and acids still seems to be ambiguous, which needs further investigation in the future. Besides the electron‐rich BPA, 2‐chlorophenol (2‐CP) and methylene blue (MB) with electron‐donating phenolic hydroxyl and/or halogen groups (for their molecular structures, see Figure S24, Supporting Information) can be also removed efficiently by Zn_SA_‐N‐C/PDS (Figure 5e). In contrast, the oxidation of ibuprofen (IBU), ciprofloxacin (CIP), and diphenhydramine (DP) containing electron‐withdrawing carboxyl groups or no electron‐donating groups seems to be less effective, confirming the electron transfer mechanism behind the Fenton‐like catalysis over Zn_SA_‐N‐C.

### 2.4 Potential Practicability of Zn_SA_‐N‐C

Benefiting from the dominant electron transfer pathway, the couple of Zn_SA_‐N‐C and PDS possess a favorable anti‐interference capability to the complicated water matrix, which accounts for the efficacious BPA elimination in the presence of prevalent environmental‐related substances and under different water systems in the above section of Fenton‐like performance evaluation. The potential practicability of Zn_SA_‐N‐C was evaluated by the treatment of actual kitchen wastewater (collected from the effluent of a shopping mall in Guangzhou city after biological treatment) and the long‐term remediation of BPA with PDS. In the three‐dimensional excitation‐emission matrix (3D‐EEM) fluorescence spectra (Figure S25, Supporting Information), the primitive actual kitchen wastewater sample exhibits two main peaks with strong fluorescence intensities assignable to aromatic fulvic‐like substances.^[^ ^44^ ^]^ After Fenton‐like reaction, the fluorescence peak intensities decreases distinctly, indicative of successful destruction of aromatic compounds in the realistic kitchen wastewater by Zn_SA_‐N‐C with PDS. By means of a homemade continuous‐flow column reactor comprising Zn_SA_‐N‐C and silica sand (Figure S26, Supporting Information), the effective BPA remediation can be attained over 216 h (Figure 5f), which attests to the admirable Fenton‐like reactivity and robust durability of Zn_SA_‐N‐C. Moreover, the diffraction pattern of the used Zn_SA_‐N‐C after reaction alters hardly (Figure S27a, Supporting Information), and meanwhile, the Zn atoms are still atomically dispersed on the surface of the spent catalyst (Figure S27b, Supporting Information), further verifying the sound stability of Zn_SA_‐N‐C in the Fenton‐like catalysis.

## 3 Conclusion

We have successfully prepared a high‐loading and low‐valence Zn single‐atom catalyst (Zn_SA_‐N‐C) with a mix‐coordination of Zn−N_4_ and Zn−N_3_ configurations for speedy Fenton‐like catalysis. Comprehensive characterizations and theoretical calculations revealed that the formation of unsaturated atomic Zn−N_3_ structure facilitates electron delocalization to reduce the average valence of Zn in the mix‐coordinated Zn−N_x_ sites so as to enhance the interaction between Zn_SA_‐N‐C and PDS. As a result, the Zn_SA_‐N‐C catalyst exhibits an admirable Fenton‐like performance in PDS activation with a superb specific activity of 0.11 L min^−1^ m^−2^, exceeding most majority of Fenton‐like SACs for PDS and even PMS activation. Experimental results unveiled that the formation of Zn_SA_‐N‐C−PDS* complex enables strong electron transfer from the electron‐rich pollutant toward the catalyst and thus dominantly realizes the rapid BPA degradation. From an application prospect, the Zn_SA_‐N‐C material achieves a remarkable long‐term remediation of BPA over 216 h using a continuous‐flow column reactor. This work offers a feasible approach to significantly increase active site density and unravels the correlation between valence state of metal species and Fenton‐like activity of SACs, which may serve as a guideline for the development of efficient and stable Fenton‐like SACs for widespread sustainable environmental applications.

## 4 Experimental Section

### Material Synthesis

The zinc single‐atom catalyst was synthesized via a surface molecule‐confined pyrolysis approach. Typically, 15 g of melamine was placed in an agate mortar, followed by the sequential addition of 2.5 mL ethanol solution of phenanthroline (1.62 g) and 10 mL ethanol solution of Zn(NO_3_)_2_·6H_2_O (1.13 g). Then, the mixture was ground uniformly for 30 min. The harvested white complex was subjected to calcination in a tube furnace at 600 °C under N_2_ atmosphere for 2 h with a heating rate of 5 °C min^−1^ to obtain the Zn‐decorated carbon nitride (Zn‐C_3_N_4_). For comparison, the Zn‐supported carbon nitride (marked as Zn Nps‐C_3_N_4_) was prepared by the same process as Zn‐C_3_N_4_ but without the addition of phenanthroline. Subsequently, the Zn‐C_3_N_4_ material was further calcined at 800°C in a tube furnace under N_2_ atmosphere for 2 h at a ramping rate of 5 °C min^−1^. After cooling naturally to room temperature, the black powder solid was immersed and stirred in 1 m H_2_SO_4_ at 60 °C for 8 h, which was collected by centrifugation and rinsed with ultrapure water for several times until neutral. The final product was dried at 60 °C and denoted as Zn_SA_‐N‐C.

### Catalytic Tests and Analyses

The catalytic performance was assessed through the oxidation of bisphenol A (BPA, a representative emerging contaminant) by Zn_SA_‐N‐C with PDS. The batch experiments were conducted in at least two duplicates. The initial solution pH (before PDS addition) was adjusted by dilute sodium hydroxide and sulfuric acid solutions. Electrochemical measurements were performed in a standard three‐electrode cell system on a CHI 700E electrochemical workstation. The identification of degradation intermediates was performed by a gas chromatography‐mass spectrometer (GC‐MS, Shimadzu GC/MS‐QP2020 NX). The long‐term stability of Zn_SA_‐N‐C was assessed via a continuous‐flow column reactor.

The other details of chemicals, characterizations, experimental procedures and theoretical computation are shown in Supporting Information.

## Conflict of Interest

The authors declare no conflict of interest.

## Supporting information

Supporting Information

## Data Availability

The data that support the findings of this study are available from the corresponding author upon reasonable request.
