# Supplementary material for: Electron Delocalization Realizes Speedy Fenton‐Like Catalysis over a High‐Loading and Low‐Valence Zinc Single‐Atom Catalyst
Source: Adv Sci (Weinh). 2023 Oct 15;10(34):2304088. doi: 10.1002/advs.202304088 (PMC10700237; doi:10.1002/advs.202304088)
Supplement: Supplementary file 1 — Supporting Information [file ADVS-10-2304088-s001.pdf]

ADVANCED SCIENCE
Open Access

Supporting Information

for *Adv. Sci.*, DOI 10.1002/advs.202304088

Electron Delocalization Realizes Speedy Fenton-Like Catalysis over a High-Loading and Low-Valence Zinc Single-Atom Catalyst

Shaosong Xin, Luning Ni, Peng Zhang, Haobin Tan, Mingyang Song, Tong Li, Yaowen Gao and Chun Hu**

Supporting Information

Electron Delocalization Realizes Speedy Fenton-Like Catalysis over a High-Loading and Low-Valence Zinc Single-Atom Catalyst

Shaosong Xin†, Luning Ni†, Peng Zhang, Haobin Tan, Mingyang Song, Tong Li, Yaowen Gao*, Chun Hu*

Institute of Environmental Research at Greater Bay, Key Laboratory for Water Quality and Conservation of the Pearl River Delta, Ministry of Education, Guangzhou University, Guangzhou 510006, China

*Corresponding author

Phone: +86-20-39346609

E-mail: gaoyw@gzhu.edu.cn; huchun@gzhu.edu.cn

The Supporting Information included:

5 Texts

4 Tables

27 Figures

31 Pages

Text S1. Chemicals

Zinc nitrate hexahydrate ($Zn(NO_3)_2$•$6H_2O$), phenanthroline, melamine (99%), sodium persulfate (PDS, $Na_2S_2O_8$, 99%), bisphenol A (BPA, ≥98%), 2-chlorophenol (2-CP, 99%), ciprofloxacin (CIP, 98%), ibuprofen (IBU, >98%), diphenhydramine (DP), methylene blue (MB), benzoquinone (BQ, ≥98%), isopropanol (IPA, 99%), ethanol (AR), sodium nitrate ($NaNO_3$, ≥99.5%), sodium chloride (NaCl, ≥99.9%), sodium bicarbonate ($NaHCO_3$, 99.5%), humic acid (HA, ≥90%), furfuryl alcohol (FFA, 99%), sulfuric acid (H_2SO_4, 96%), 5,5-dimethyl-1-pyrroline N-oxide (DMPO, ≥98%), 2,2,6,6-tetramethyl-4-piperidinol (TMP , 98%), dimethyl sulfoxide (DMSO) were purchased from Adamas Reagent Co., Ltd. Other chemicals were used as received. All solutions were prepared using ultrapure water (Milli-Q, 18.2 MΩ cm) from a purification system.

Text S2. Catalytic Tests and Analyses

The catalytic performance was assessed by adding Zn_{SA}-N-C (0.1 g L^{-1}) into 40 mL of bisphenol A (BPA) solution (0.1 mM) under magnetic stirring in a constant temperature-controlled water bath at 30 °C. After pre-interaction for 30 min to ensure the uniform suspension, PDS (2 mM) was injected into the suspension to initiate the Fenton-like reaction. At predetermined time intervals, 1 mL aliquots of reaction solution were withdrawn by syringe and immediately filtered through a Millipore filter (0.22 μm) for analysis. The batch experiments were carried out in at least two duplicates. The initial solution pH (before PDS addition) was adjusted by dilute sodium hydroxide and

sulfuric acid solutions. The long-term durability of Zn_{SA}-N-C was evaluated via a homemade continuous-flow column reactor.

The concentration of BPA was measured by a 1260 Infinity HPLC (Agilent, USA) with a UV detector and a Poroshell 120 EC-C18 column (4.6 × 100 mm, 2.7 μm) at a detection wavelength of 225 nm. A mixture of methanol/water (70:30, v/v) was used as a mobile phase, and the flowing rate was set as 1.0 mL min^{-1}. The total organic carbon (TOC) concentration was determined by a TOC-L analyzer (Shimadzu, Japan). The amount of zinc ions leaching from the catalyst after catalytic reaction was measured by inductively coupled plasma optical emission spectrometry (ICP-OES) (Avio 200, PerkinElmer). The PDS concentration was analyzed with spectrophotometric method on a Hach DR 6000 UV-vis spectrometer. Measurement of residual sulfate anion (SO_4^{2-}) was conducted by a Thermo ICS-600 ion chromatography (IC). Electron paramagnetic resonance (EPR) measurements for in situ detection of hydroxyl ($^{\bullet}OH$)/sulfate ($SO_4^{\bullet-}$) and superoxide ($O_2^{\bullet-}$) radicals were undertaken on a Bruker A300 spectrometer with 5,5-dimethyl-1-pyrroline N-oxide (DMPO) as the spin-trapping agent in the aqueous and methanol media, respectively. Singlet oxygen ($^1O_2$) was detected by EPR spectrometry with the spin-trapping agent of 2,2,6,6-tetramethyl-4-piperidinol (TMP) in aqueous solution. The generation of $O_2^{\bullet-}$ was also investigated via a nitroblue tetrazolium (NBT) method. Electrochemical measurements including cyclic voltammetry (CV), open-circuit potential (OCP), and chronoamperometry (CP) were conducted in a standard three-electrode cell system on a CHI 700E electrochemical workstation with a sodium sulfate solution (0.5 M) as an electrolyte. The as-prepared

samples, Pt foil and Ag/AgCl electrode were utilized as working electrode, counter electrode and reference electrode, respectively. The CV analysis was conducted at a scan rate of 50 mV s^{-1}. For volatile product identification, 50 mL of the sample was extracted with 1.5 mL of dichloromethane, and the extract was analyzed on a gas chromatography-mass spectrometer (GC-MS, Shimadzu GC/MS-QP2020 NX) in EI mode using a full scan range of 40-400 m/z S8 (DB-1701 column, 30 m × 0.25 mm × 0.25 μm; injector 280 °C, oven 50 °C held for 5 min, and then ramped to 250 °C at 5 °C min^{-1}, and auxiliary 270 °C). The in situ diffuse reflectance infrared Fourier transform spectroscopy (DRIFTS) analysis of BPA degradation over Zn_{SA}-N-C via PDS activation was performed on a PerkinElmer Frontier FTIR spectrometer.

Text S3. Characterizations

The morphology of and elemental distribution of samples were observed by a FEI Quanta FEG 250 scanning electron microscopy (SEM) and a FEI Tecnai G2 F20 transmission electron microscopy (TEM) equipped with an energy dispersive X-ray (EDX) spectroscopy. The atomic dispersion of zinc atoms on the surface of catalyst was analyzed on an aberration-corrected high-angle annular dark-field scanning transmission electron microscopy (HAADF-STEM, FEI Themis Z). The phase structures of samples were investigated using a Bruker D8 Advance diffractometer with Cu Kα radiation (λ = 1.54178 Å). The N_2 adsorption/desorption isotherm measurements were performed by a Micrometrics ASAP 2460 apparatus at 77K. Raman and in situ Raman spectra were recorded on a LabRAM HR Evolution (HORIBA, France) with a 532 nm excitation laser. X-ray photoelectron spectroscopy (XPS) analysis was carried

out on a Thermo ESCALAB 250 Xi instrument with monochromatic Al Kα radiation. The zinc content of sample was measured by inductively coupled plasma optical emission spectrometry (ICP-OES, Avio 200, PerkinElmer). Fourier transform infrared (FTIR) spectra were acquired with a Nicolet iS10 spectrometer with samples dispersed in KBr at a resolution of 4 cm^{-1}. X-ray absorption fine structure (XAFS) spectroscopy analysis was conducted in a transmission mode at beamline 14W1B of the Shanghai Synchrotron Radiation Facility (SSRF). Data processing of the X-ray absorption near edge structure (XANES) and extended X-ray absorption fine structure (EXAFS) spectra were carried out using the IFEFFIT package. The solid-state EPR spectra of catalysts were collected from a Bruker A300 spectrometer.

Text S4. Galvanic Oxidation System (GOS) Experiment

For the preparation of Zn_{SA}-N-C-coated graphite electrode, 5 mg of catalyst powder was added into a mixture of Nafion solution (50 μL) and absolute ethanol (2.95 mL) under sonication for 30 min. Then, the suspension (3 mL) was pipetted onto both sides of graphite sheets and the process was repeated once to ensure an even coating of the catalyst. A commercial salt bridge was used to connect the two half cells and retain electrical neutrality during the measurement. A tinned wire was employed to connect the two electrodes for electron transfer.

Text S5. Theoretical Computations

All calculations were performed using the DMol^3 code base on the spin-unrestricted density function theory (DFT).[1] The electronic exchange and correlation effects were descried by the generalized gradient approximation (GGA) with the Perdew-Burke-

Ernzerhof (PBE) function.[2] The Grimme's methods were added in order to describe the van der Waals interactions. The All Electron Relativistic was employed to include all electrons explicitly and introduce some relativistic effects into the core. Furthermore, the double numerical plus polarization (DNP) basis set was selected. A smearing of 0.005 Ha of the orbital occupation was applied to speed up electronic convergence. The real-space global orbital cutoff radius was selected as 4.6Å for the sake of high-quality results. The convergence tolerance of energy, maximum force and displacement were 1×10^{-5} Ha, 0.002 Ha $Å^{-1}$ and 0.005 Å, respectively. The formation energy (E_f) was defined as follows:[3]

$$E_f = E_{ZnN_xC_y} - x\mu_N - \mu_{Zn} - y\mu_C \quad \text{(S1)}$$

where $E_{ZnN_xC_y}$ is the total energy of Zn–N_x moiety; μ is the chemical potential of the corresponding species. The reference states are chosen to be perfect graphene for C, nitrogen molecule for N, and isolated zinc atom for Zn.

Table S1. Elemental composition of the as-prepared sample by XPS and ICP-OES.

Sample	Elemental content			
	C (at%)	N (at%)	Zn (at%)	Zn (wt%)
Zn_{SA}-N-C	69.51	27.45	3.04	11.54

Table S2. Structure parameters of samples extracted from the EXAFS fitting of Zn K-edge using ZnPc as the $Zn–N_4$ core structure.

Sample	Model	*CN*	*R* (Å)	σ^2 (Å^2)	ΔE_0 (eV)	*R*-factor
ZnPc molecule	ZnPc	3.9	2.00	0.006	2.98	0.016
Zn_{SA}-N-C	ZnPc	3.6	1.96	0.004	0.56	0.017

Table S3. Structure parameters of Zn_{SA}-N-C extracted from the EXAFS fitting of Zn K-edge using the $Zn–N_3$ core structure according to DFT calculations (see Figure S8a for its atomic structure).

Sample	Model	*CN*	*R* (Å)	σ^2 (Å^2)	ΔE_0 (eV)	*R*-factor
Zn_{SA}-N-C	$Zn–N_3$	2.1	1.71	0.004	6.09	0.021

Note: C*N* is coordination number; *R* is interatomic distance (the bond length between Zn central atoms and surrounding N coordination atoms); σ^2 is Debye-Waller factor to account for both thermal and structural disorders; ΔE_0 is inner potential correction; *R* factor indicates the goodness of the fit.

Table S4. Specific activity comparison of Zn_{SA}-N-C with recently reported transition-metal (Fe, Co, Mn, Cu)-based SACs for the Fenton-like reaction.

Catalyst (dosage, g L^{-1})	S_{BET} (m^2 g^{-1})	Pollutant (mg L^{-1})	Persulfate concentration (g L^{-1})	Removal efficiency	Rate constant (min^{-1})	Specific activity (L min^{-1} m^{-2})	Ref
SAFe-75-N-C (0.1)	1196.48	CAP (32.3)	PDS (0.24)	93.1% (480 min)	–	–	[4]
Co_{SA}-N_3-C (0.05)	380.03	BPA (11.4)	PDS (0.48)	100% (4 min)	1.280	0.067	[5]
DFeNC (0.1)	495.94	SMX (5.0)	PDS (0.24)	100% (90 min)	0.070	0.0014	[6]
Fe/Cu-N-C (0.1)	664.8	CAP (20.0)	PDS (1.20)	90.8% (20 min)	0.093	0.0014	[7]
Cu_{SA}-NC (0.04)	758.7	2,4-DCP (16.3)	PDS (0.12)	100% (30 min)	0.284	0.009	[8]
Cu_1/NG (0.1)	–	BPA (20.0)	PDS (0.12)	100% (6 min)	1.40	–	[9]
FeSA-NEPBC (0.04)	609.1	BPS (5.0)	PDS (0.10)	100% (40 min)	0.153	0.006	[10]
$Fe_{0.4}Cu_{0.6}$-N-C (0.4)	31.56	TBBPS (5.0)	PDS (0.48)	98.7% (60%)	0.06	0.005	[11]
Zn_{SA}-N-C (0.1)	222.3	BPA (22.8)	PDS (0.48)	100% (3 min)	2.435	0.110	**This work**
SA-Zn-NC (0.1)	232.13	AO7 (17.5)	PMS (0.06)	98% (30 min)	0.136	0.006	[12]
Fe-N_4-PC-2 (0.3)	25.0	BPA (10)	PMS (0.10)	100% (10 min)	0.80	0.107	[13]
FeN_x-C-600 (0.2)	135.2	BPA (20)	PMS (0.15)	98% (15 min)	0.357	0.013	[14]
Fe_{SA}-N/C-20 (0.15)	522.3	BPA (20)	PMS (0.40)	100% (20 min)	0.318	0.004	[15]
Fe_1/CN (0.5)	435.0	BPA (10)	PMS (0.30)	100% (10 min)	0.55	0.002	[16]
Fe-N-O-GC (0.1)	209.7	BPA (10)	PMS (0.12)	100% (30 min)	0.20	0.009	[17]
SA-Fe/CN (0.02)	945.2	BPA (20)	PMS (0.06)	97% (5 min)	0.54	0.028	[18]
Fe-CN_x (0.05)	320.0	BPA (20)	PMS (0.10)	94% (8 min)	0.44	0.027	[19]
SA-Fe-NC (0.05)	288.3	BPA (23)	PMS (0.60)	100% (3 min)	1.99	0.138	[20]

Fe_{SA}-N-C-20 (0.15)	586.9	BPA (20)	PMS (0.15)	99% (30 min)	0.24	0.004	[21]
FeCo-NC (0.1)	375.0	BPA (20)	PMS (0.20)	100% (4 min)	1.252	0.033	[22]
Co-SAs (0.2)	345.4	BPA (200)	PMS (0.60)	82% (12 min)	0.157	0.002	[23]
Co–N_2 (0.2)	880.4	BPA (11)	PMS (0.60)	100% (5 min)	0.695	0.004	[24]
Co-N-CNTs (0.1)	178.5	SMX (10)	PMS (0.30)	100% (16 min)	0.157	0.008	[25]
Mn-ISAs@CN (0.2)	1159.2	BPA (20)	PMS (0.20)	100% (6 min)	1.138	0.005	[26]
SA-Cu/rGO (0.1)	34.3	SMX (10)	PMS (0.40)	99.6% (40 min)	0.087	0.025	[27]
Cu-SA (0.1)	1102.0	BPA (23)	PMS (0.15)	60% (5 min)	0.839	0.007	[28]

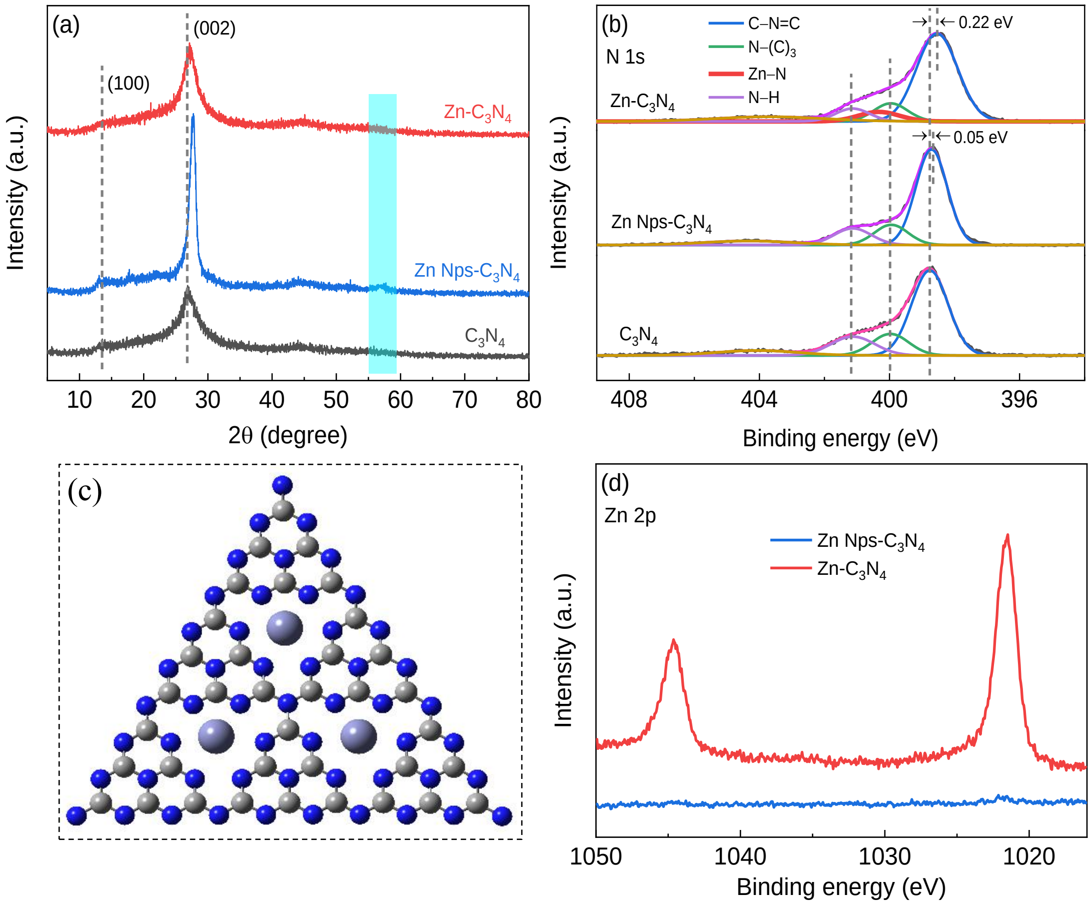

Figure S1. (a) XRD patterns and (b) XPS N 1s spectra of C_3N_4 and Zn-based C_3N_4. (c) Atomic structure of Zn-C_3N_4. (d) XPS Zn 2p spectra of Zn-based C_3N_4. Cyan, blue, and gray spheres represent Zn, N, and C atoms, respectively.

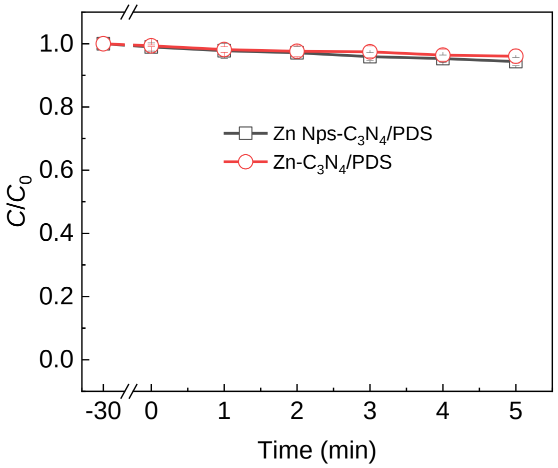

Figure S2. Removal of BPA by Zn-based C_3N_4 with PDS. Reaction conditions: [BPA] = 100 μM; [catalyst] = 0.1 g L^{-1}; [PDS] = 2 mM; [Temp] = 30 °C; initial pH = 6.7.

To ascertain the importance of phenanthroline for sequestering the Zn species, the Zn-supported carbon nitride (denoted as Zn Nps-C_3N_4) was prepared by the same process as Zn-C_3N_4 but without the addition of phenanthroline. X-ray diffraction (XRD) patterns (**Figure S1a**) show that both Zn Nps-C_3N_4 and Zn-C_3N_4 samples maintain the basic heterocyclic skeleton of C_3N_4, where the peaks at approximately 13.6° and 26.8° correspond to the (100) and (002) planes regarding the in-plane repeating units of continuous heptazine framework and the stacking of conjugated aromatic structure, respectively.[29] Compared with pristine C_3N_4, the (100) peak in Zn Nps-C_3N_4 shifts negatively, while the (002) peak presents an obvious positive shift (by ~0.9°) with an evidently increased intensity. Besides, a new weak peak emerges at approximately 56.9° in Zn Nps-C_3N_4 assignable to the metallic ZnO.[30] These alternations illuminate that the Zn aggregation occurs in Zn Nps-C_3N_4 during the calcination process without the

addition of phenanthroline, which results in the distortion in the in-plane aromatic stacking to decrease the interlamellar distance of C_3N_4 and facilitates the crystallization of C_3N_4.[31] By contrast, the (100) peak almost vanishes in Zn-C_3N_4 upon adding phenanthroline as a starting material, and meanwhile, the (002) peak shifts slightly toward the higher angle. More importantly, no identifiable metallic Zn phase can be detected in Zn-C_3N_4. This result confirms the crucial role of phenanthroline for securing and secluding the Zn atoms in the course of calcination, conducive to the confinement of Zn species in the electron-rich cavities of C_3N_4 containing macrocyclic units with pyridinic N. In this regard, the X-ray photoelectron spectroscopy (XPS) analyses of C_3N_4, Zn Nps-C_3N_4, and Zn-C_3N_4 were performed. By comparing the XPS N 1s spectrum of C_3N_4 with that of Zn Nps-C_3N_4, and Zn-C_3N_4 (**Figure S1b**), the C–N=C peak shifts toward the lower binding energy slightly (by 0.05 eV) and significantly (by 0.22 eV) for Zn Nps-C_3N_4 and Zn-C_3N_4, respectively. In addition, a novel sub-peak appears at approximately 400.32 eV in Zn-C_3N_4 referring to the Zn–N bond, while this peak is absent in Zn Nps-C_3N_4. The apparent negative shift of the C–N=C peak and the appearance of Zn–N peak in Zn-C_3N_4 manifest the binding of Zn and pyridinic N within the cavities of the heptazine units,[32] where the atomic structure of Zn-C_3N_4 can be observed in **Figure S1c**. Moreover, the Zn content of Zn-C_3N_4 was determined to be 3.24 at% by XPS, which is significantly higher that of Zn Nps-C_3N_4 (0.19 at%), as evidenced by the much stronger Zn 2p peak intensity for Zn-C_3N_4 relative to that for Zn Nps-C_3N_4 (**Figure S1d**). Unfortunately, both Zn Nps-C_3N_4 and Zn-C_3N_4 exhibit limited Fenton-like activity in PDS activation (**Figure S2**). This situation may be

ascribed to the insufficient electronic structure modulation and the inadequate dosage of C_3N_4-based materials or PDS, which are not the focus of the present work. It therefore necessitates the conversion of Zn-C_3N_4 into a nitrogen-doped carbon-based Zn single-atom catalyst. Upon a higher temperature pyrolysis at 800 °C under N_2 atmosphere, the Zn-C_3N_4 can be converted to a high-loading Zn SAC. The above results highlight the advantage of the surface molecule-confined calcination method for the preparation of the Zn SAC, namely Zn_{SA}-N-C, with a high Zn loading up to 11.54 wt%.

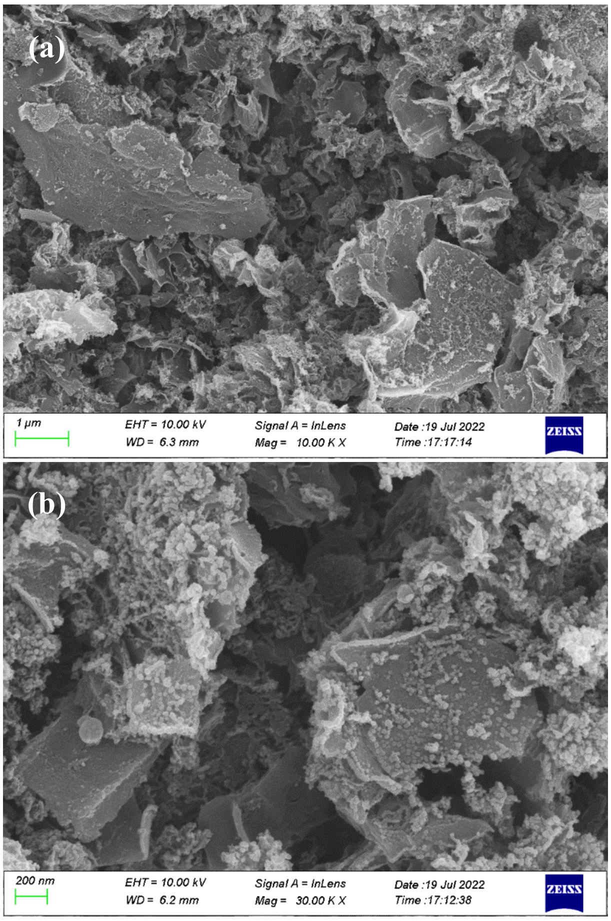

Figure S3. SEM images of Zn_{SA}-N-C.

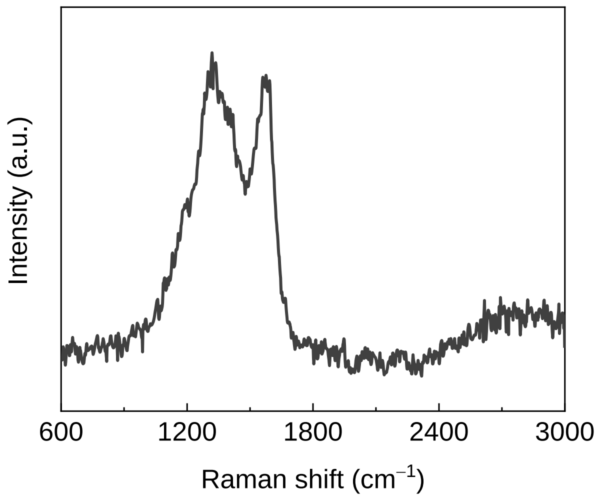

Figure S4. Raman spectrum of Zn_{SA}-N-C.

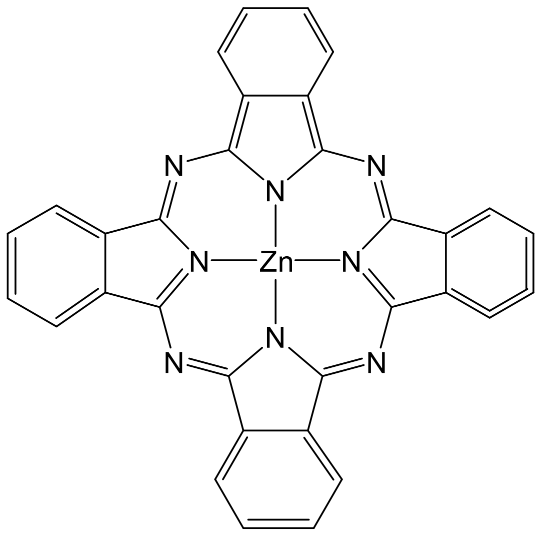

Figure S5. Molecular structure of ZnPc.

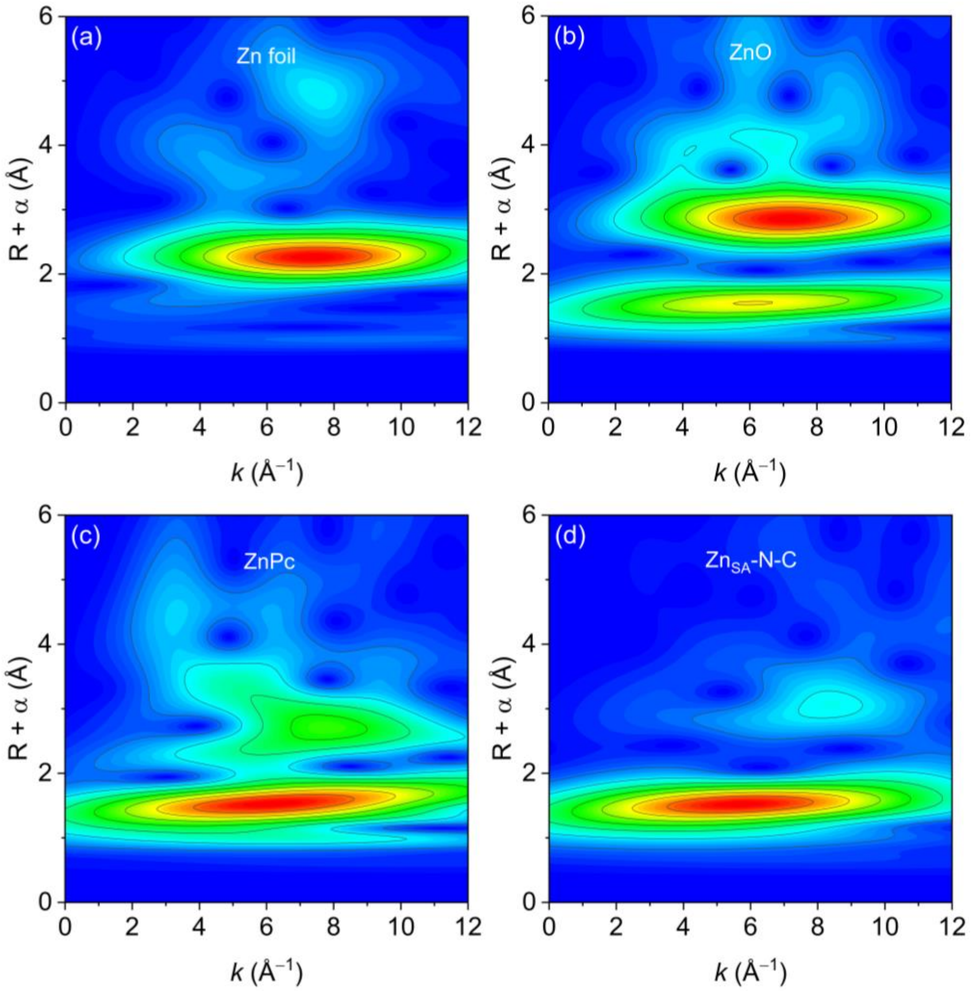

Figure S6. Wavelet transform (WT) spectra of (a) Zn foil, (b) ZnO, (c) ZnPc, and (d) Zn_{SA}-N-C samples.

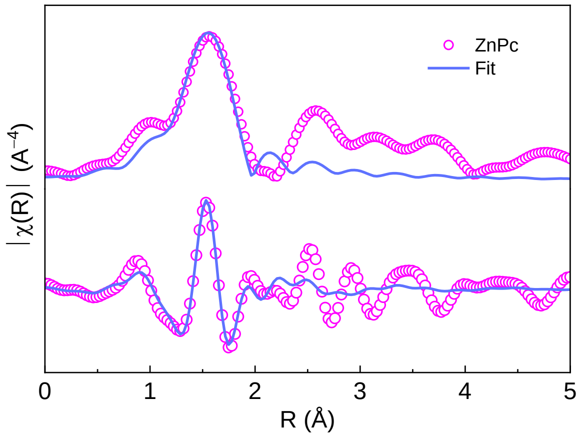

Figure S7. EXAFS fitting curves of the ZnPc sample using ZnPc as a typical Zn–N_4 core structure in R space.

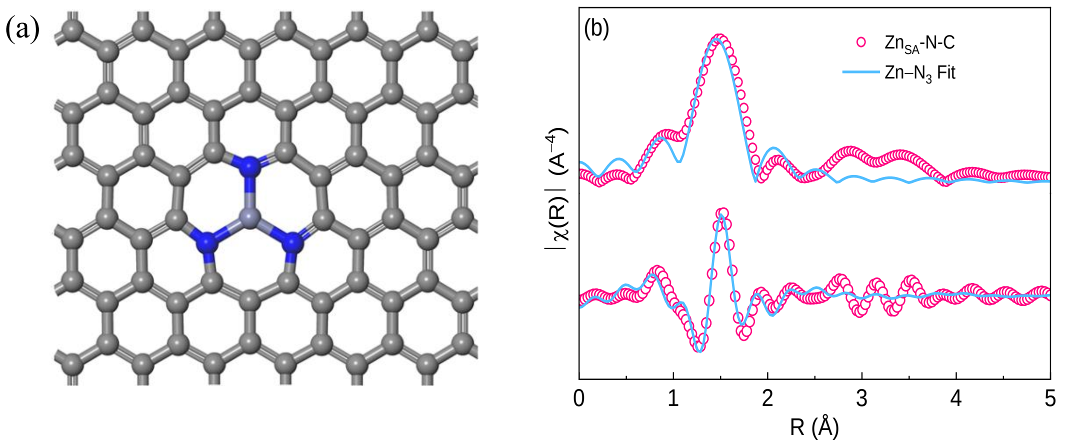

Figure S8. (a) Model structure of Zn–N_3 simulated from DFT calculations (cyan, blue, and gray spheres represent Zn, N, and C atoms, respectively). (b) EXAFS fitting curves of Zn_{SA}-N-C using the simulated Zn–N_3 core structure in R space.

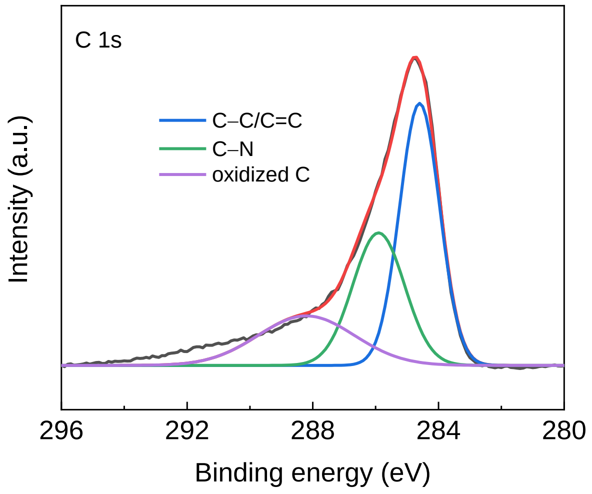

Figure S9. XPS C 1s spectrum of Zn_{SA}-N-C.

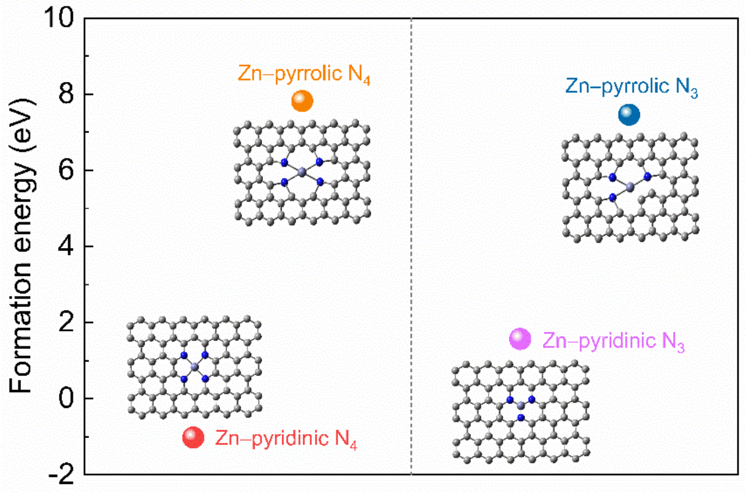

Figure S10. Formation energy comparison of different Zn–N_x configurations. Cyan, blue, and gray spheres represent Zn, N, and C atoms, respectively.

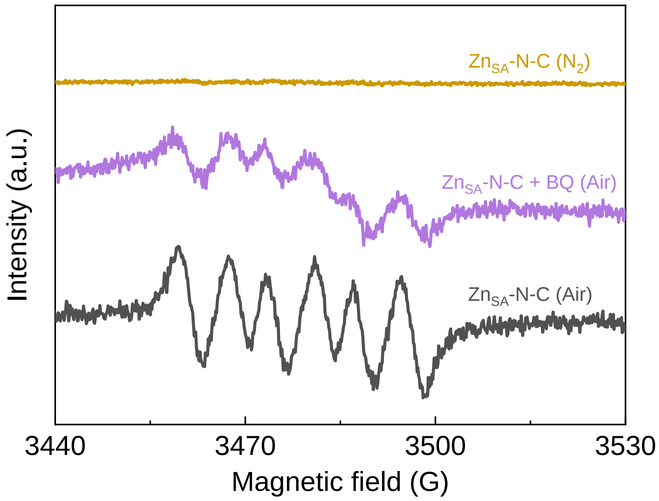

Figure S11. EPR spectra for $O_2^{\bullet-}$ of Zn_{SA}-N-C under different conditions. Reaction conditions: [BQ] = 5 mM.

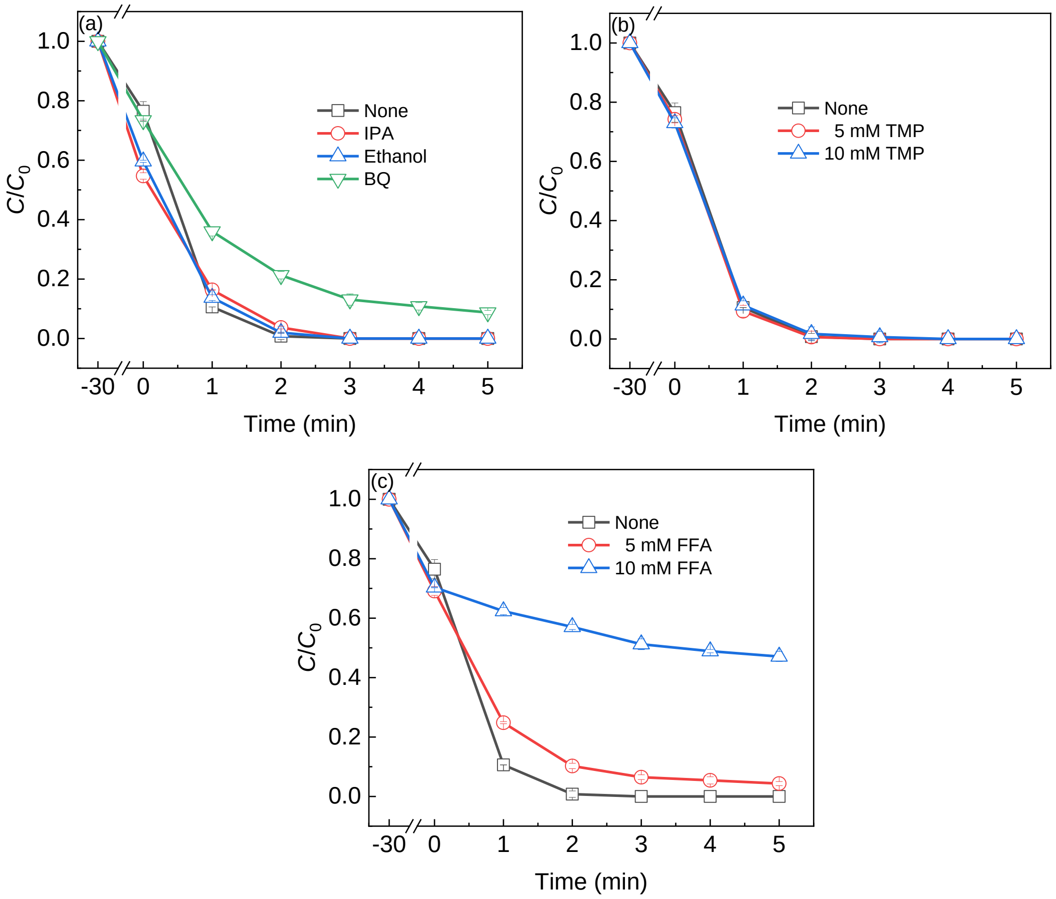

Figure S12. Effect of (a) various scavengers, (b) TMP and (c) FFA with different concentrations on BPA removal by Zn_{SA}-N-C/PDS. Reaction conditions: [BPA] = 100 μM; [Zn_{SA}-N-C] = 0.1 g L^{-1}; [PDS] = 2 mM; [Temp] = 30 °C; [IPA] = [Ethanol] = 500 mM; [BQ] = 5 mM; initial pH = 6.7 for (a). [BPA] = 100 μM; [Zn_{SA}-N-C] = 0.1 g L^{-1}; [PDS] = 2 mM; [Temp] = 30 °C; initial pH = 6.7 for (b) and (c).

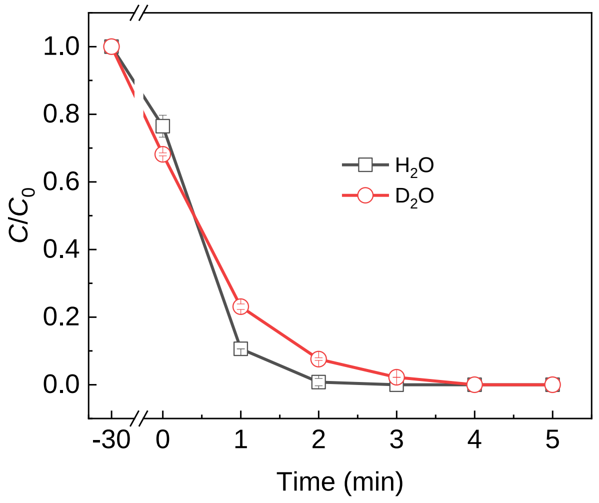

Figure S13. BPA removal by Zn$_{SA}$-N-C/PDS in D_2O. Reaction conditions: [BPA] = 100 μM; [Zn$_{SA}$-N-C] = 0.1 g L^{-1}; [PDS] = 2 mM; [Temp] = 30 °C; initial pH = 6.7.

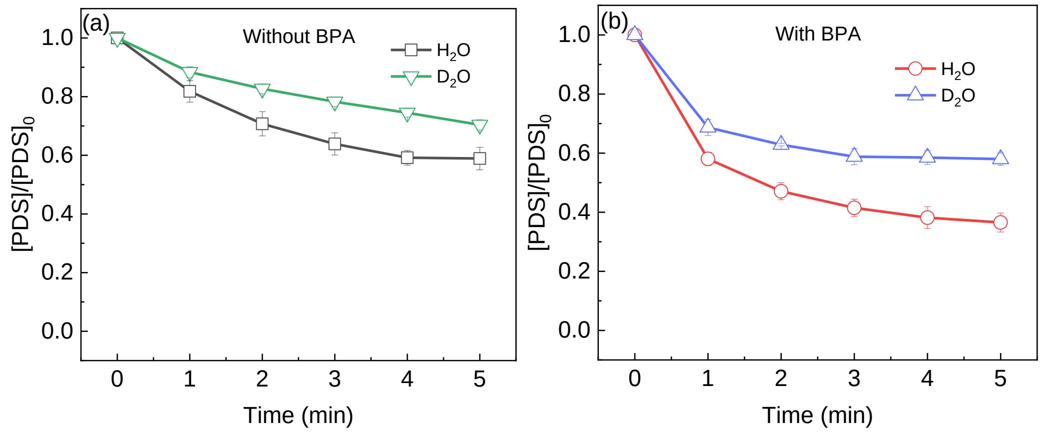

Figure S14. Decay of PDS over Zn$_{SA}$-N-C in H_2O and D_2O (a) without and (b) with BPA. Reaction conditions: [BPA] = 100 μM; [Zn$_{SA}$-N-C] = 0.1 g L^{-1}; [PDS] = 2 mM; [Temp] = 30 °C; initial pH = 6.7. (if any)

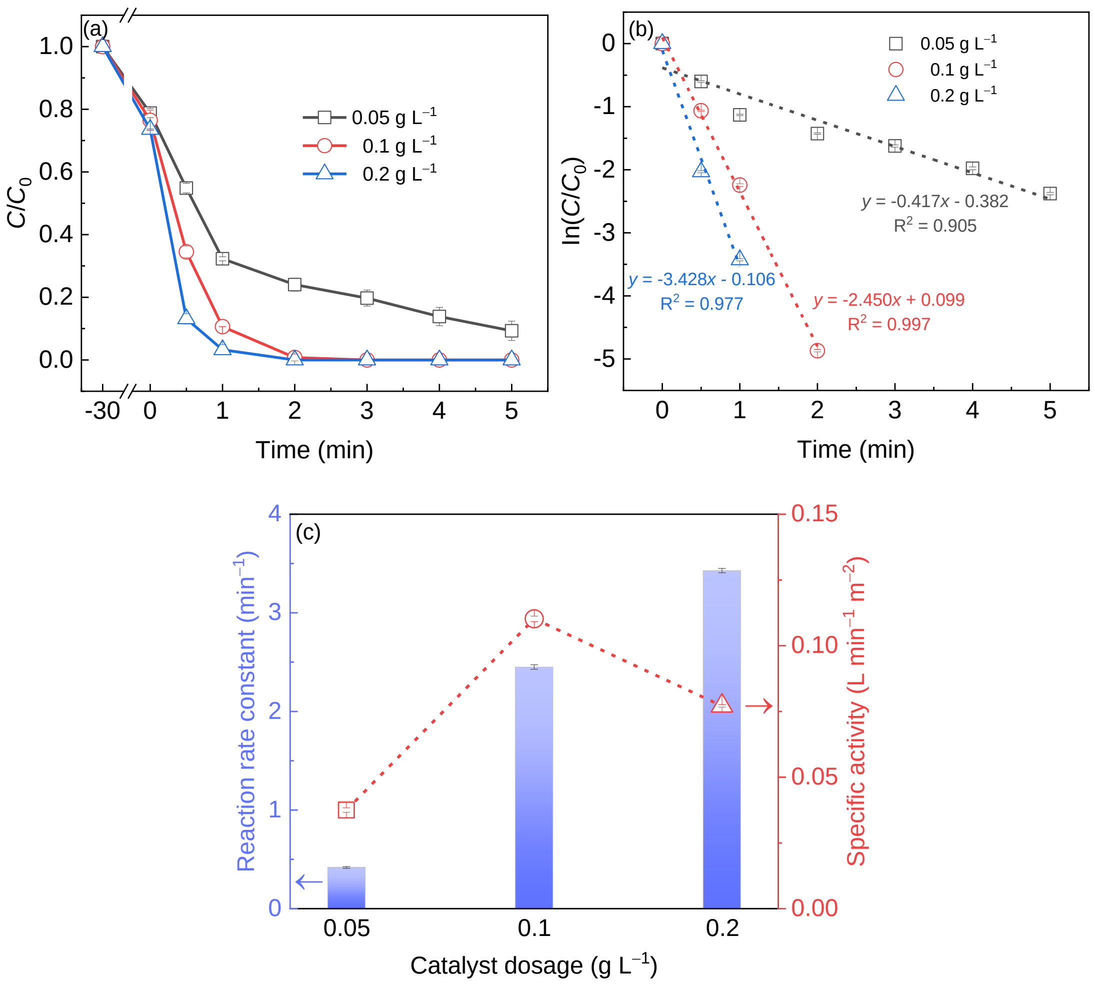

Figure S15. (a) Effect of catalyst dosage on BPA removal by Zn_{SA}-N-C with PDS. (b) Pseudo-first-order kinetics of BPA degradation by Zn_{SA}-N-C/PDS. (c) Comparison of reaction rate constant and specific activity at different catalyst dosages. Reaction conditions: [BPA] = 100 μM; [PDS] = 2 mM; [Temp] = 30 °C; initial pH = 6.7.

Considering the achievement of complete BPA degradation by Zn_{SA}-N-C/PDS within 2 min at the catalyst dosage of 0.2 g L^{-1}, an extra liquid sample was withdrawn at the reaction time of 0.5 min to favor the calculation of the relevant reaction rate constants.

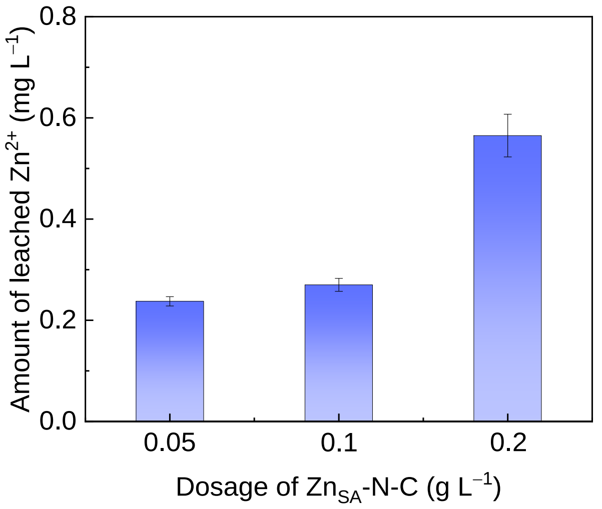

Figure S16. Amount of leached Zn^{2+} at different dosage of Zn_{SA}-N-C. Reaction conditions: [BPA] = 100 μM; [PDS] = 2 mM; [Temp] = 30 °C; initial pH = 6.7.

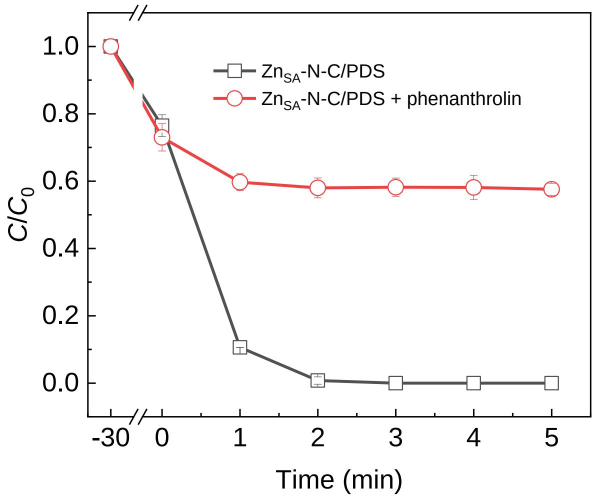

Figure S17. Effect of phenanthrolin on BPA removal by Zn_{SA}-N-C/PDS. Reaction conditions: [BPA] = 100 μM; [Zn_{SA}-N-C] = 0.1 g L^{-1}; [PDS] = 2 mM; [phenanthrolin] = 10 mM; [Temp] = 30 °C; initial pH = 6.7.

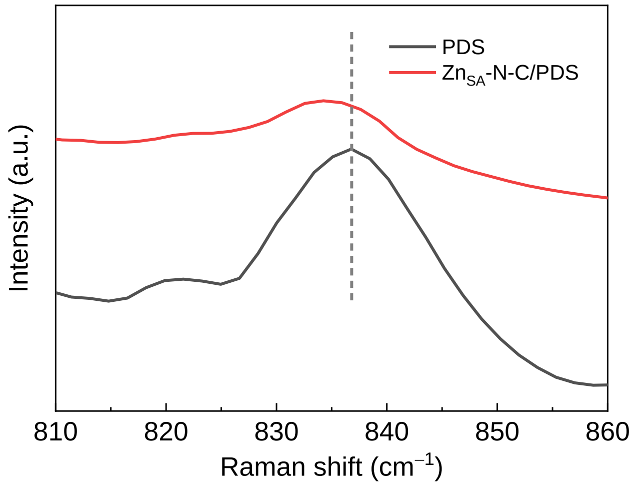

Figure S18. In situ Raman spectra of Zn$_{SA}$-N-C after interaction with PDS at 836 cm^{-1}.

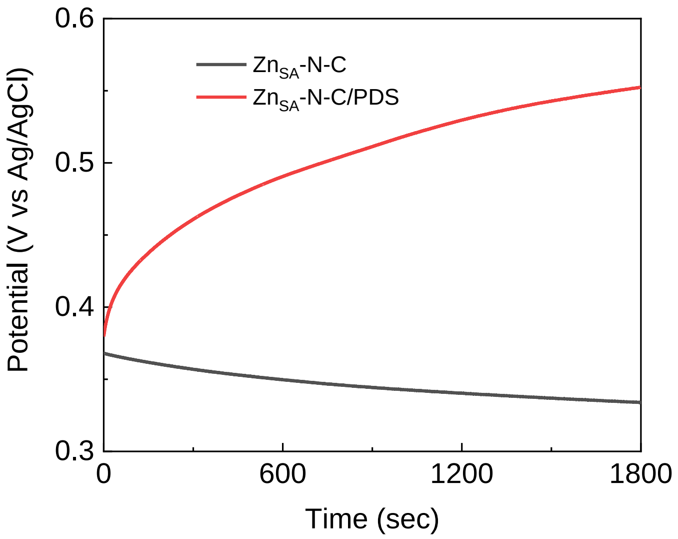

Figure S19. Open-circuit potential curves of Zn$_{SA}$-N-C with and without PDS.

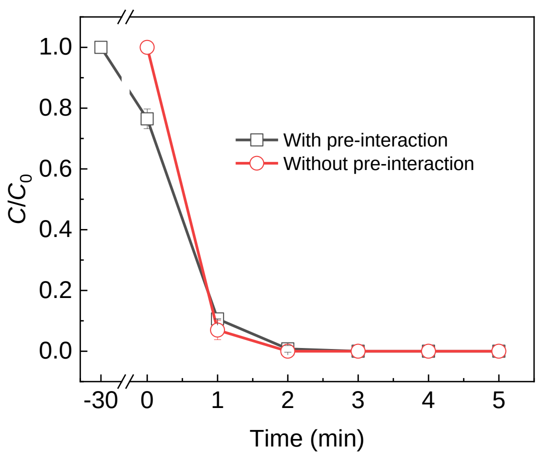

Figure S20. Removal of BPA by Zn$_{SA}$-N-C/PDS with and without pre-interaction of Zn$_{SA}$-N-C and BPA. Reaction conditions: [BPA] = 100 μM; [Zn$_{SA}$-N-C] = 0.1 g L^{-1}; [PDS] = 2 mM; [Temp] = 30 °C; initial pH = 6.7.

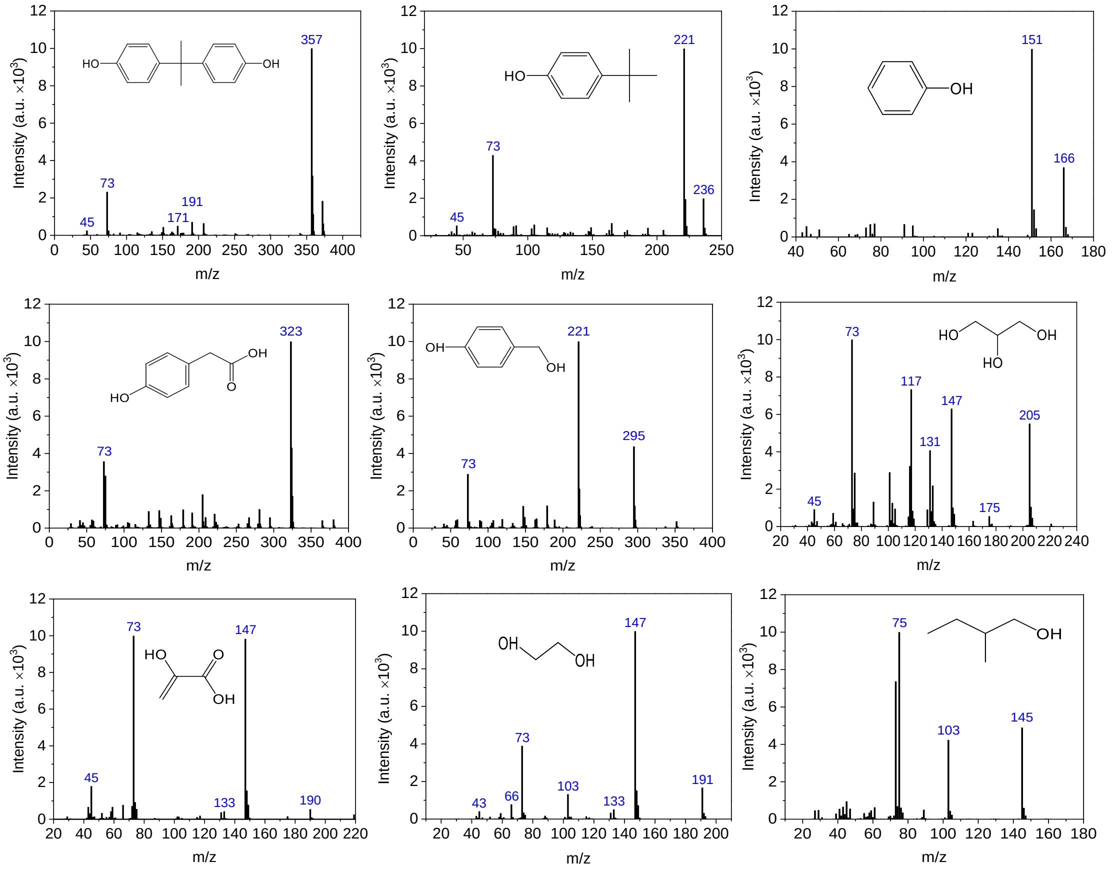

Figure S21. MS spectra of intermediates from BPA degradation by Zn$_{SA}$-N-C and Zn$_{SA}$-N-C/PDS.

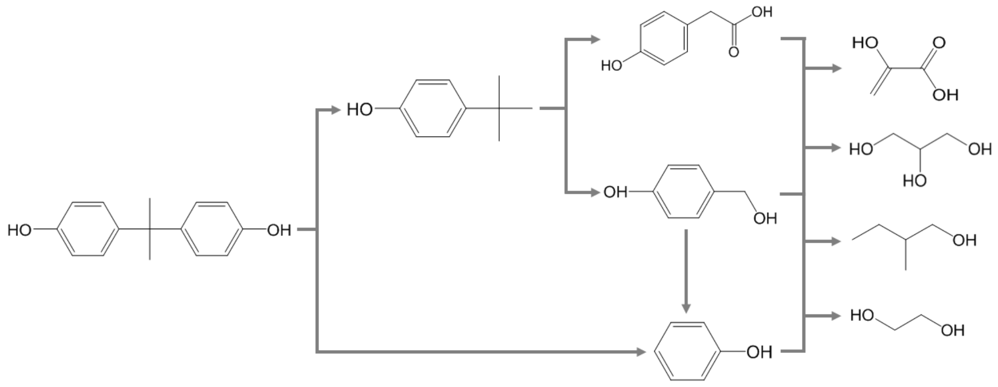

Figure S22. Possible BPA oxidation pathway over Zn_{SA}-N-C via PDS activation.

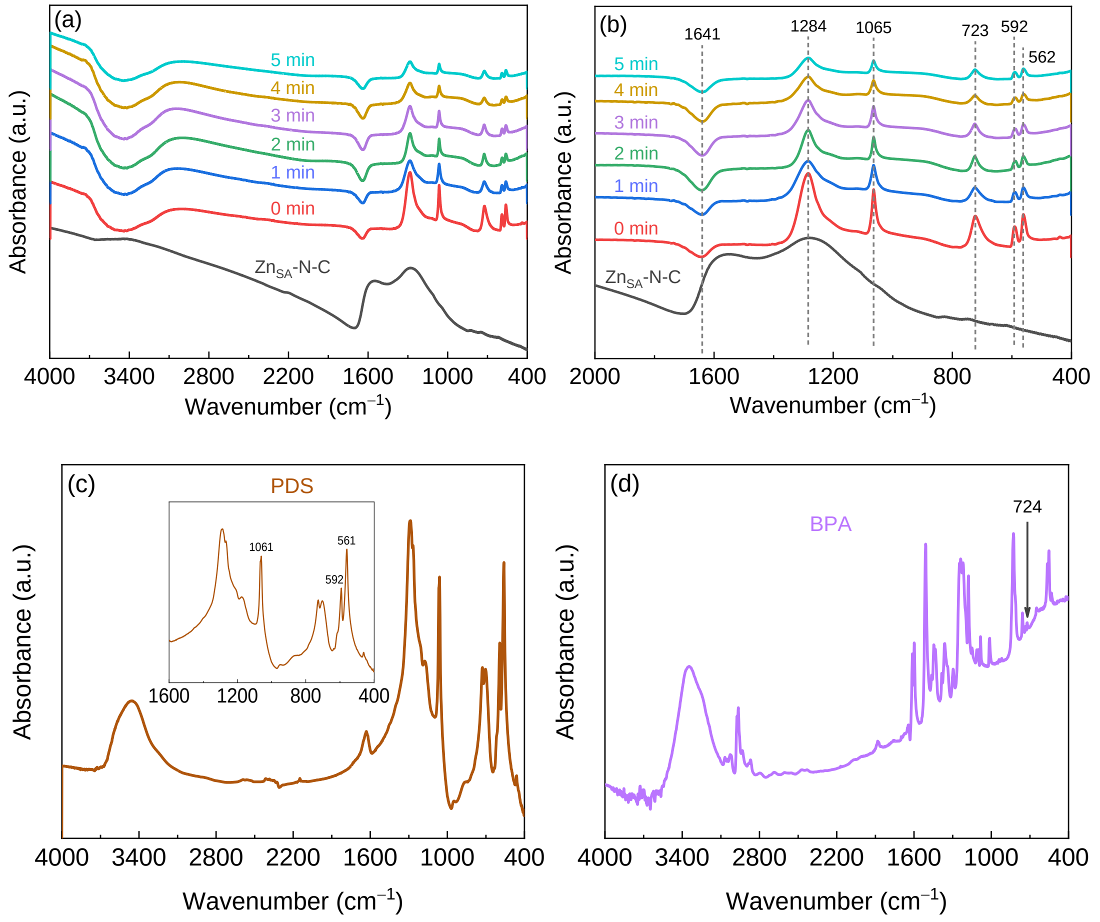

Figure S23. In situ DRIFT spectra of BPA degradation over Zn_{SA}-N-C via PDS activation in (a) full absorption and (b) relatively low wavenumber regions. FTIR spectra of (c) PDS and (d) BPA molecules.

In the spectrum of Zn_{SA}-N-C before reaction (**Figure S23a**), two peaks emerge at 1279 and 1553 cm^{-1}, which can be accredited to the C−N and/or C=N stretching vibrations, confirming the incorporation of N atoms into the carbon frameworks of Zn_{SA}-N-C. In the in situ DRIFTS spectra, a broad peak appears at approximately 3450 cm^{-1} in all samples, assignable to the −OH stretching vibration from the adsorbed free H_2O on the surface of the catalyst. Besides, several peaks can be noted in the relatively low wavenumber absorption regions. From **Figure S23b**, the peaks locating at 562, 592, and 1065 cm^{-1} correspond to the vibrations of S−O and/or S=O bonds in the PDS molecule, as evidenced by the FTIR spectrum of PDS in **Figure S23c**. The peak at 723 cm^{-1} refers to the out-of-plane bending vibration of C−H bond within phenolic compounds (see the spectrum of BPA in **Figure S23d**). Besides, the appearance of strong peak at 1284 cm^{-1} can be caused by the stretching vibration of C−O/O-H bond in alcohols. The negative peak at 1641 cm^{-1} is associated with the vibration of carboxylic C=O bond.[33] As the reaction proceeds, those peaks at the wavenumber less than 1300 cm^{-1} undergo the gradual decrease in their intensities. By contrast, the intensity of peak at 1641 cm^{-1} increases firstly and then decreases progressively with the reaction time. These phenomena demonstrate (i) the adsorption of PDS and BPA molecules onto Zn_{SA}-N-C alongside their successive decomposition and (ii) the formation of intermediates from BPA degradation including carboxylic acids and alcohols together with their further oxidation in the Zn_{SA}-N-C/PDS system.

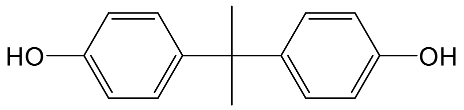

Bisphenol A (BPA)

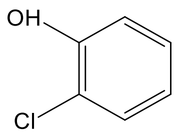

2-chlorophenol (2-CP)

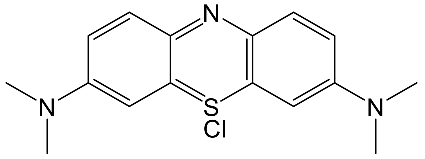

Methylene blue (MB)

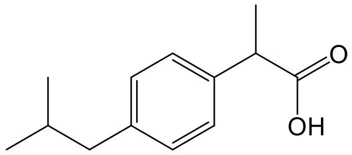

Ibuprofen (IBU)

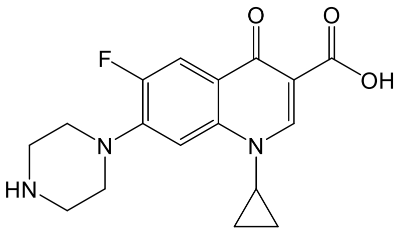

Ciprofloxacin (CIP)

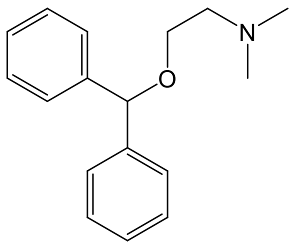

Diphenhydramine (DP)

Figure S24. Molecular structures of various organic pollutants.

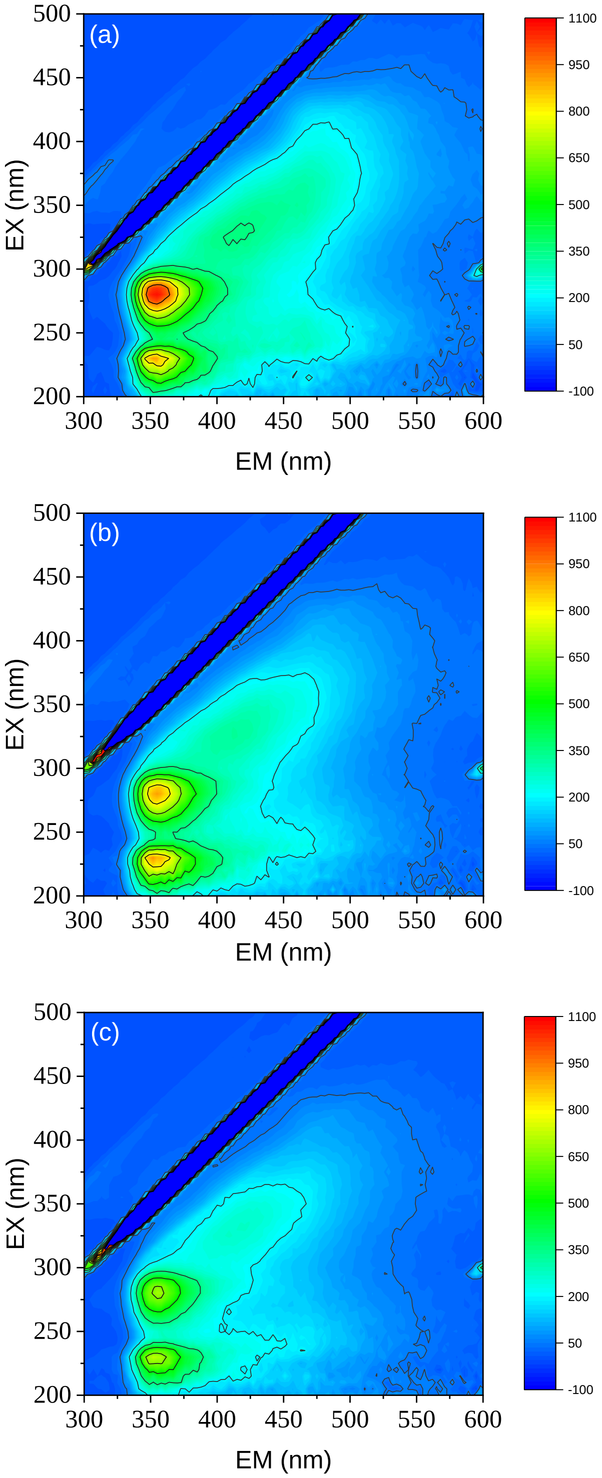

Figure S25. 3D-EEM fluorescence spectra of actual kitchen wastewater (a) before reaction, and after reaction by (b) Zn_{SA}-N-C for 30 min and (c) Zn_{SA}-N-C/PDS for 5 min. Reaction conditions: [Zn_{SA}-N-C] = 0.1 g L^{-1}; [PDS] = 2 mM; [Temp] = 30 °C.

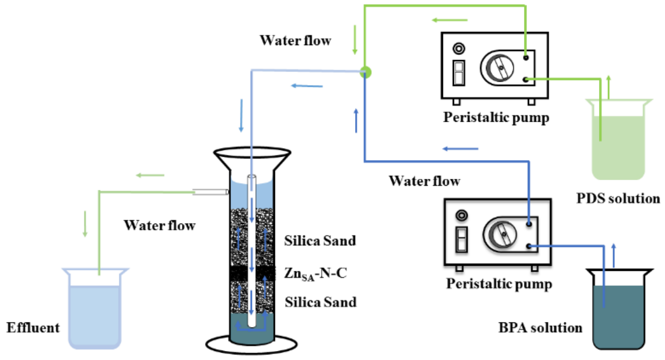

Figure S26. Schematic diagram of the continuous-flow reactor comprising Zn$_{SA}$-N-C-filled column with silica sand.

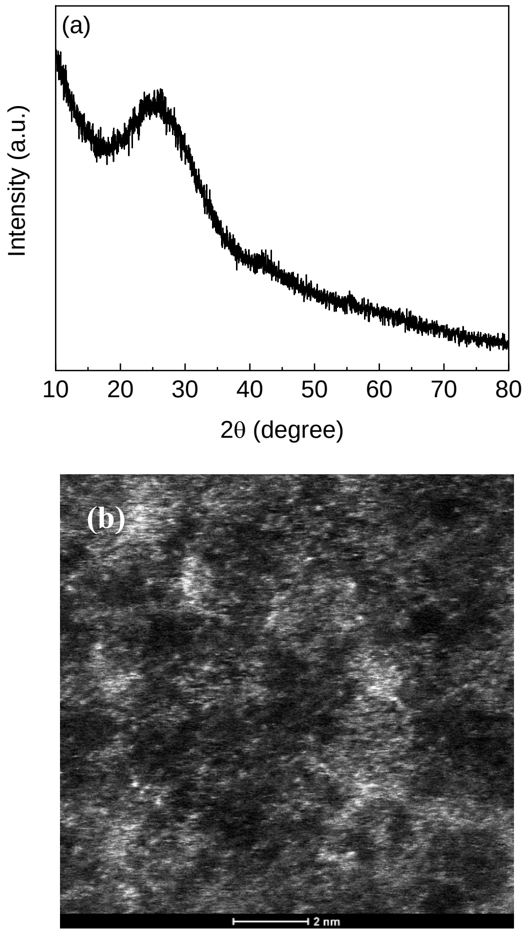

Figure S27. (a) XRD pattern and (b) HAADF-STEM image of used Zn$_{SA}$-N-C.

References

[1] G. Kresse, J. Furthmuller, *Phys. Rev. B* **1996**, *54*, 169-186.

[2] J. P. Perdew, K. Burke, M. Ernzerhof, *Phys. Rev. Lett.* **1996**, *77*, 3865-3868.

[3] K. Liu, G. Wu, G. Wang, *J. Phys. Chem. C* **2017**, *121*, 11319-11324.

[4] N. Du, Y. Liu, Q. Li, W. Miao, D. Wang, S. Mao, *Chem. Eng. J.* **2021**, *413*, 127545.

[5] X. Liang, D. Wang, Z. Zhao, T. Li, Z. Chen, Y. Gao, C. Hu, *Appl. Catal. B: Environ.* **2022**, *303*, 120877.

[6] Y. Shang, X. Liu, Y. Li, Y. Gao, B. Gao, X. Xu, Q. Yue, *Chem. Eng. J.* **2022**, *446*, 137120.

[7] H. Wu, J. Yan, X. Xu, Q. Yuan, J. Wang, J. Cui, A. Lin, *Chem. Eng. J.* **2022**, *428*, 132611.

[8] F. Li, Z. Lu, T. Li, P. Zhang, C. Hu, *Environ. Sci. Technol.* **2022**, *56*, 8765-8775.

[9] B. Wang, C. Cheng, M. Jin, J. He, H. Zhang, W. Ren, J. Li, D. Wang, Y. Li, *Angew. Chem. Int. Ed.* **2022**, *61*, e202207268.

[10] Z. Wang, J. Bao, H. He, S. Mukherji, L. Luo, J. Du, *Chem. Eng. J.* **2023**, *458*, 141513.

[11] Q. Wang, D. Zhou, C. Liu, X. Chen, L. Liu, K. Lin, *Chem. Eng. J.* **2023**, *460*, 140681.

[12] Z. Zhao, H. Tan, P. Zhang, X. Liang, T. Li, Y. Gao, C. Hu, *Angew. Chem. Int. Ed.* **2023**, *62*, e202219178.

[13] J. Wang, B. Li, Y. Li, X. Fan, F. Zhang, G. Zhang, W. Peng, *Adv. Sci.* **2021**, *8*, 2101824.

[14] B. Zhang, X. Li, K. Akiyama, P. A. Bingham, S. Kubuki, *Environ. Sci. Technol.* **2022**, *56*, 1321-1330.

[15] T. Yang, S. Fan, Y. Li, Q. Zhou, *Chem. Eng. J.* **2021**, *419*, 129590.

[16] L. S. Zhang, X. H. Jiang, Z. A. Zhong, L. Tian, Q. Sun, Y. T. Cui, X. Lu, J. P. Zou, S. L. Luo, *Angew. Chem. Int. Ed.* **2021**, *60*, 21751-21755.

[17] S. Wang, L. Xu, J. Wang, *Environ. Sci. Technol.* **2021**, *55*, 15412-15422.

[18] Y. Xiong, H. Li, C. Liu, L. Zheng, C. Liu, J. O. Wang, S. Liu, Y. Han, L. Gu, J.Qian, D. Wang, *Adv. Mater.* **2022**, *34*, 2110653.

[19] W. Miao, Y. Liu, D. Wang, N. Du, Z. Ye, Y. Hou, S. Mao, K. Ostrikov, *Chem. Eng. J.* **2021,** *423*, 130250.

[20] Y. Gao, Y. Zhu,;T. Li, Z. Chen, Q. Jiang, Z. Zhao, X. Liang, C. Hu, *Environ. Sci. Technol.* **2021,** *55*, 8318-8328.

[21] Y. Li, T. Yang, S. Qiu, W. Lin, J. Yan, S. Fan, Q. Zhou, *Chem. Eng. J.* **2020**, *389*, 124382.

[22] X. Li, X. Huang, S. Xi, S. Miao, J. Ding, W. Cai, S. Liu, X.Yang, H. Yang, J.Gao, J. Wang, Y. Huang, T. Zhang, B. Liu, *J. Am. Chem. Soc.* **2018**, *140*, 12469-12475.

[23] Y. Gao, C. Yang, M. Zhou, C. He, S. Cao, Y. Long, S. Li, Y. Lin, P. Zhu, C. Cheng, *Small* **2020**, *16*, 2005060.

[24] X. Liang, D. Wang, Z. Zhao, T. Li, Y. Gao, C. Hu, *Adv. Funct. Mater.* **2022,** *32*, 2203001.

[25] J. Miao, Y. Zhu, J. Lang, J. Zhang, S. Cheng, B. Zhou, L. Zhang, P. J. J. Alvarez, M. Long, *ACS Catal.* **2021**, *11*, 9569-9577.

[26] J. Yang, D. Zeng, Q. Zhang, R. Cui, M. Hassan, L. Dong, J. Li, Y. He, *Appl. Catal. B: Environ.* **2020**, *279*, 119363.

[27] F. Chen, X. L. Wu, L. Yang, C. Chen, H. Lin, J. Chen, *Chem. Eng. J.* **2020**, *394*, 124904.

[28] S. Zuo, Z. Guan, F. Yang, D. Xia, D. Li, *J. Mater. Chem. A* **2022**, *10*, 10503-10513.

[29] Y. Gao, Y. Zhu, L. Lyu, Q. Zeng, X. Xing, C. Hu, *Environ. Sci. Technol.* **2018**, *52*, 14371-14380.

[30] S. Zhan, H. Zhang, X. Mi, Y. Zhao, C. Hu, L. Lyu, *Environ. Sci. Technol.* **2020**, *54*, 8333-8343.

[31] W. D. Oh, V. W. C. Chang, Z. T. Hu, R. Goei, T. T. Lim, *Chem. Eng. J.* **2017**, *323*, 260-269.

[32] Z. Zhao, P. Zhang, H. Tan, X. Liang, T. Li, Y. Gao, C. Hu, *Small* **2023**, *19*, 2205583.

[33] K. Li, X. Fang, Z. Fu, Y. Yang, I. Nabi, Y. Feng, A. U. R. Bacha, L. Zhang, *J. Hazard. Mater.* **2020**, *398*, 123007.
